# Applying implementation science frameworks to identify factors that influence the intention of healthcare providers to offer PrEP care and advocate for PrEP in HIV clinics in Colombia: a cross-sectional study

**DOI:** 10.1186/s43058-022-00278-2

**Published:** 2022-03-16

**Authors:** Jorge Luis Martinez-Cajas, Julian Torres, Hector Fabio Mueses, Pilar Camargo Plazas, Marcela Arrivillaga, Sheila Andrea Gomez, Ximena Galindo, Ernesto Martinez Buitrago, Beatriz Eugenia Alvarado Llano

**Affiliations:** 1 Division of Infectious Diseases, Department of Medicine, Queen’s University, Kingston, ON K7L 3 N6 Canada; 2 Montefiore Medical Center, Moses Division, Albert Einstein College of Medicine, The Oval Center at Montefiore, 3230 Bainbridge Ave, Bronx, NY 10467 USA; 3 Corporación de Lucha Contra el Sida, Carrera 56 2- 120, Cali, Colombia; 4 School of Nursing, Queen’s University, Kingston, ON K7L 3 N6 Canada; 5 Facultad de Ciencias de la Salud, Pontificia Universidad Javeriana Cali, Colombia, Calle, 18 118-250 Cali, Colombia; 6 Departamento de Medicina Interna, Universidad del Valle, Calle 5 36-08 Hospital Universitario del Valle, Cali, Colombia; 7 Public Health Science, Queens University, Kingston, ON K7L 3 N6 Canada

**Keywords:** Implementation science, HIV PrEP, Theoretical domains framework, Consolidated framework for implementation research

## Abstract

**Background:**

Few studies have used implementation science frameworks to identify determinants of PrEP prescription by healthcare providers. In this work, we developed and psychometrically examined a questionnaire using the theoretical domains framework (TDF) and the consolidated framework for implementation research (CFIR). We used this questionnaire to investigate what factors influence the intention of healthcare providers to offer PrEP care and advocate for PrEP.

**Methods:**

We conducted a cross-sectional study in 16 HIV healthcare organizations in Colombia. A 98-item questionnaire was administered online to 129 healthcare professionals. One hundred had complete data for this analysis. We used exploratory factor analysis to assess the psychometric properties of both frameworks, and multinomial regression analysis to evaluate the associations of the frameworks’ domains with two outcomes: (1) intention to offer PrEP care and (2) intention to advocate for PrEP impmentation.

**Results:**

We found support for nine indices with good internal consistency, reflecting PrEP characteristics, attitudes towards population needs, concerns about the use of PrEP, concerns about the role of the healthcare systems, knowledge, beliefs about capabilities, professional role, social influence, and beliefs about consequences. Notably, only 57% of the participants were likely to have a plan to care for people in PrEP and 66.7% were likely to advocate for PrEP. The perception of the need for PrEP in populations, the value of PrEP as a practice, the influence of colleagues, and seeing PrEP care as a priority was related to being less likely to be unwilling to provide or advocate for PrEP care.

**Conclusion:**

Our findings suggested the importance of multilevel strategies to increase the provision of PrEP care by healthcare providers including adquisition of new skills, training of PrEP champions, and strength the capacity of the health system.

**Supplementary Information:**

The online version contains supplementary material available at 10.1186/s43058-022-00278-2.

Contributions to literature

- There are few studies using implementation science frameworks (CFIR and TDF) to develop questionnaires to assess factors related to the intention of providing PrEP in health care professionals. We found nine scales assessing domains or subdomains of the CFIR and TDF, and we found consistent associations between those scales and intention to offer PrEP care and advocacy for PrEP in health professionals.
- The determinants of intention to either provide PrEP or advocate for PrEP were multifactorial and go beyond PrEP attitudes and perceptions, which are the most common findings in the literature. The perception of the need for PrEP in populations, the value of PrEP as a practice, the influence of colleagues, and seeing PrEP care as a priority was related to being less likely to be unwilling to provide or advocate for PrEP care.
- Using implementation science frameworks allowed the identification of domains that have previously rarely been addressed in the literature on PrEP and that could help devise strategies to change PrEP adoption and advocacy by health care professionals.

## Introduction

At the end of 2017, an estimated 1.8 million people were living with HIV in Latin America, and men who have sex with men (MSM) and transgender women (TGW) are disproportionately affected. In 2020, 180,000 people were living with HIV in Colombia, of whom 9300 were new cases that year [1]. The prevalence of HIV infection in MSM and TGW has remained high at around 10%, with little change over the last 15 years. Further, approximately 61% of people living with HIV (PLH) in Latin America and 64% in Colombia are on antiretroviral therapy [1]. HIV pre-exposure prophylaxis (PrEP) has been strongly recommended for people at high risk of acquiring HIV in Latin America, including MSM and TGW (2), and the combination of tenofovir disoproxil fumarate and emtricitabine (TDF/FTC) to prevent HIV infection was approved by the Colombian drug regulatory agency INVIMA in 2019. Yet, at the the time of this study, this approval has not been widely advertised to healthcare providers (HCP) nor has it been covered by drug plans. In contrast, Brazil approved and made TDF/FTC available in the healthcare system for PrEP in 2017 [2, 3]. In Peru, Mexico, and Colombia, demonstration projects have been conducted, but TDF/FTC coverage has been limited only to project participants. Implementation studies in Latin America have focused on PrEP feasibility, safety, and adherence (2), and the evidence remains scarce on HCP willingness and intention to offer PrEP, PrEP-related services and on individual and contextual factors that affect their intention to offer PrEP care.

With an implementation science perspective in mind, our main objective was to explore the determinants of intention to offer PrEP care and advocate for PrEP in HCP. In Colombia, only physicians can prescribe antiretroviral drugs, including PrEP [4]. Therefore, the outcome *intention to prescribe PrEP medications* cannot be used to explore the support or rejection of this intervention in non-physicians. Thus, we chose PrEP advocacy as an alternative outcome that allows us to assess the support, or lack of, for PrEP in non-physicians. Targeting non-physicians is relevant since PrEP provision relies on a multidisciplinary intervention where physicians and non-physicians coordinate care [5]. We targeted all HCP working in the HIV-specialized clinics, including social workers, psychologists, pharmacists, nurses, and physicians because (1) both HIV care and PrEP require the use of similar practices such as HIV testing and counseling, medication adherence, and assessment of HIV transmission/acquisition risk and (2) HIV clinics in Colombia already function with HIV care teams that involve all these health care disciplines. Importantly, care team-based approaches and high interprofessional collaboration has been associated with better HIV care and improvements in counseling and linkage to services in PrEP users [6, 7].

Two implementation science theories were selected to develop a questionnaire and identify appropriate targets for interventions to promote the implementation of PrEP in Colombia. The first is the *consolidated framework for implementation research* (CFIR), which can assess determinants of current practices, potential barriers, facilitators to practice change, and implementation of new strategies [8]. The CFIR is composed of five domains: *intervention characteristics*, *outer/inner setting*, *characteristics of individuals*, and *process*. A recent publication recommends the inclusion of a sixth domain, *the characteristics of healthcare systems* [9]. The CFIR posits that the adoption of any intervention or program requires an understanding of determinants at multiple levels and multilevel strategies. This is the case for the adoption of PrEP, where it is recognized that care and adoption should include identifying determinants at the individual (i.e., community members and providers), organizational (clinics, centers, community organizations), and contextual levels (i.e., policies, stigma, social inequities) [5, 10–13].

All domains of the CFIR (and its subdomains), except the one related to the process domain, were selected to guide the construction of a questionnaire and a guide for semi-structured interviews [14]. Table 1 displays how domains were handled in this study. Various studies have found relationships between the four domains of the CFIR analyzed here and PrEP adoption. For instance, Piper et al. described that family planning clinics with higher readiness to implement PrEP had HCP who valued PrEP and HIV prevention, believed that PrEP was compatible with their patient population, perceived that PrEP was a low complexity intervention, and had leadership supporting that intervention [10]. Sullivan et al. found that the perceptions of HCP about PrEP characteristics (e.g., effectiveness, relative advantage, simplicity), the lower capacity of the health system, the lack of policies for Medicaid expansion, and peers norms were important barriers for PrEP adoption [12]. Pinto et al. described system barriers such as funding, access, and mistrust to PrEP adoption by HCP and uptake by potential users [11]. We have previously described similar health system barriers [15].

**Table 1:** CFIR and TDF domains relevant for PrEP implementation and those selected to build the questionnaire

	Definition	Included in the survey	Relevant theme in qualitative analysis
**I. Intervention characteristics**			
Evidence strength and quality	HCPs’ perceptions of the quality and validity of evidence support the belief that PrEP will have desired outcomes	Yes	Yes
Relative advantage	HCPs’ perception of the advantage of implementing PrEP versus an alternative solution	Yes	Yes
Adaptability	The degree to which PrEP can be adapted, tailored, refined, or reinvented to meet local needs	Yes	Yes
Complexity	Perceived difficulty of implementation, reflected by duration, scope, radicalness, disruptiveness, centrality, and intricacy and number of steps required to implement	Yes	Yes
Cost	Costs of PrEP and costs associated with implementing PrEP include investment, supply, and opportunity costs	Yes	Yes
**II. Outer setting**			
Patient needs and resources	The extent to which people’s need for PrEP is recognized by HCP and barriers and facilitators to meet those needs	Yes	Yes
Cosmopolitanism	The degree to which the clinics are networked with other external organizations	No	Yes
Peer pressure	Pressure from community organizations ot other external organizations to implement PrEP	No	Yes
External policies and incentives	Social, political and economic influences over PrEP implementation	No	Yes
**III. Health systems**			
Attitudes	Attitudes of HCP regarding the preparedness of the health system to implement PrEP	Yes	Yes
Concerns	Concerns of HCP regarding the preparedness of the health system to implement PrEP	Yes	Yes
System architecture	The administrative design of Colombian health system or interacting systems that may influence PrEP implementation	No	Yes
Funding priorities	Manager’s perception regarding the degree to which funding agent preferences and priorities influence implementation	No	Yes
Available resources	The level of resources at health system level needed for implementation of PrEP	No	Yes
**IV. Characteristics of individuals**			
Knowledge	HCP are aware of PrEP as an HIV prevention strategy and are familiar with the delivery of PrEP components	Yes	Yes
Beliefs about capabilities/self-efficacy	The self-confidence of HCP in performing activities related to PrEP and implementing PrEP	Yes	No
Professional role/compatibility	The extent to which PrEP implementation will be/is perceived by HCP as part of their work or responsibilities or compatible with their work	Yes	Yes
Social influences	Peer opinions about PrEP that may influence the implementation of PrEP	Yes	No
Control	HCP perceptions that they have control over the decision to offer PrEP care	Yes	No
Individual stage of change	HCP intentions to offer PrEP care or advocate for PrEP in the clinic	Yes	No
Beliefs about consequences	It refers to HCP beliefs about the value of PrEP, consequences, rewards or incentives for managing people in PrEP	Yes	Yes
**V Inner setting**			
Structural characteristics	The social architecture, age, maturity, and size of the clinics	No	Yes
Networks and communications	The nature and quality of webs of social networks and the nature and quality of formal and informal communications within the clinics	No	Yes
Culture	Norms, values, and basic assumptions of each clinic	No	Yes
Tension for change	The degree to which managers/clinic directors perceive the current situation as intolerable or needing change	No	Yes
Compatibility	The degree how PrEP fits with existing workflows	No	Yes
Relative priority	Manager perception of the importance of the implementation within the organization	No	Yes
Readiness for implementation	Tangible and immediate indicators of organizational commitment to its decision to implement PrEP	No	Yes
Leadership engagement	Commitment, involvement, and accountability of leaders and managers with the implementation of PrEP	No	Yes
Available resources	The level of resources at clinical level needed for implementation of PrEP	No	Yes
Access to knowledge and information	Ease of access to information and knowledge about PrEP and how to incorporate it into work tasks	No	Yes
**VI Process**			
Planning	Existence of any plan to implement PrEP in the clinic	No	Yes
Engaging	Strategies to engage populations at risk and other leaders in PrEP	No	Yes
Executing	Experience of implementation of PrEP	No	No
Evaluating	Evaluation of implementation of PrEP	No	No

The second theory we applied was the theoretical domains framework (TDF), which permits a deeper exploration of the *characteristics of individuals* not fully achieved by the CFIR [16] The TDF is a model of implementation science that provides a broad view of cognitive, affective, social, and environmental influences on practices or behaviors [16, 17]. TDF has been used to design interventions and conduct process evaluations. In this analysis, the TDF informs us about individual barriers that need to be addressed to promote change in the HCP’s intentions. A recent systematic review synthesized the evidence [18] and other publications underlined the utility of the TDF to understand the HCP’s intention to offer PrEP care and to successfully implement PrEP [19, 20]. Among the key barriers for PrEP provision, studies have identified elements of the TDF domains including a lack of knowledge [21] or skills (e.g., familiarity with counseling) [22], fear of drug resistance, concerns about the possibility of compensatory reduction of condom use, medication cost [23, 24], the social influence of peers on the HCP’s willingness to prescribe PrEP [11], and the lack of clinical guidelines [20]. Of the 14 domains of TDF, except for optimism and behavioral regulation, 12 were found to be relevant and were used to build our questionnaire . Seven of them are analyzed in this work (Table 1).

In this work, we present the process of development of new scales to assess barriers and facilitators for the provision of PrEP care and advocacy for PrEP by HCP which were based on the CFIR and TDF domains. We also examined the validity of these scales and describe their distribution in a sample of HIV care providers in Colombia. And finally, we examined the relationship between the CFIR/TDF domains or subdomains with the providers’ intention to offer PrEP care and advocate for PrEP implementation. Identification of the determinants at the HCP level will inform the design of behavioral and implementation strategies that can be tailored to the Colombian context.

## Methods

### Design

We conducted a cross-sectional study on HIV-care providers using an online platform from August to December of 2019. The study was completed with the support of the VIHCOL (Red de Clinicas de VIH en Colombia) network. This network of HIV providers comprises 16 specialized HIV care centers (of the 75 available in Colombia) in 18 Colombian cities and intends to improve the care of people living with HIV through research and education. The clinics that participated in this study offered services to individuals of all health coverage plans (see details in supplementary file # 1a) and collectively served around 24,000 people living with HIV in Colombia at the time this study was conducted [14]. Importantly, this study was conducted before the first PrEP demonstration project in Colombia was started. This study adhere to STROBE reccomendations (see Recommendation # 1b in Additional file 2)

### The PrEP-COL study

The PrEP-COL study was funded by the Colombian Ministry of Science in March 2019 and aimed to address the gap in PrEP implementation by identifying barriers and facilitators at the healthcare system, health care professionals, and the community. Our main purpose was to inform the implementation of PrEP in HIV-specialized health care centers. The PrEP-COL study uses three levels of analysis: HIV care providers, the clinics and their managers, and the populations at risk of HIV acquisition. It was conceived with a mixed-method design (1) to assess facilitators of awareness of and intention to use PrEP in MSM and TGW, (2) to identify intentions and determinants of intention to offer or advocate for PrEP in health care providers (HCP), and (3) to identify the clinics leadership/managers’ intention to implement PrEP and its determinants. Methodological details are accessible elsewhere [25].

### Recruitment of participants

We presented this project to VIHCOL representatives who were asked to share this information with all HCP at their clinics. The research team contacted clinic coordinators who provided us with the email addresses of individuals who expressed their willingness to participate. All who agreed to be part of the study (*N* = 338) were contacted via email and invited to complete the online survey, which was expected to take 20–30 min. Three reminders were sent 15 days apart, and no financial contribution was offered to participants.

### Survey development

We started by creating a list of items that could measure each domain of the CFIR and TDF. For the CFIR items, our preliminary list was extracted and modified from (1) Henderson et al. implementation scales [26], which includes intervention characteristics (complexity, compatibility, and relative advantage) and adopter characteristics (concerns, attitudes, and self-efficacy), (2) a literature review on PrEP perceptions among HCPs [27], and (3) the CFIR questions tool [28]. Items related to TDF were derived or modified from (1) existing published literature of other clinical practices that used the TDF [29], (2) the TDF questionnaire of Huijg et al. and other similar studies [30, 31], and (3) questionnaires available online that address factors related to PrEP adoption [32]. An initial set of 98 items was drafted by one of the authors with training in implementation science models (BEA). Subsequently, three infectious disease specialists familiar with PrEP and bilingual in Spanish and English (JLM, JT, and EM) reviewed the list of items to verify duplicability, relevance, and clarity. All corrections were incorporated into a new version and reviewed again by the remaining authors, who are familiar with both the development of questionnaires and HIV care in Colombia (BEA, HFM, MA). We then uploaded the questionnaire to the Qualtrics platform, assessed its functionality, and conducted two additional rounds of feedback. Cognitive interviews were then conducted with three physicians, a social worker, and a nurse, all experienced in HIV care and knowledgeable of PrEP. The cognitive interviews were analyzed using thematic analysis with codes representing each aspect of the questionnaire that needed adjustments in either language, organization, clarity, or comprehension. Cognitive interviews were conducted by a research assistant with experience in qualitative interviews and analysis and one of the authors (HFM). The cognitive interviews were audio-recorded and used to inform final adjustments. The items included in this analysis are available in Table 1 in supplementary file #3. The full questionnaire in Spanish is available in supplementary file #4.

### Validity of the scales

Exploratory factor analysis was conducted to assess the unidimensionality of each of the domains and subdomains (supplementary file #5). Nine scales with good reliability were found. Four scales summarized CFIR domains or sub-domains: *characteristics of PrEP*, *attitudes towards population needs*, *concerns of the use of PrEP in populations*, and *concerns about the healthcare system*. The other five scales summarized the domain *characteristics of individuals* built using TDF constructs: *knowledge, beliefs about capabilities*, *professional role*, *social influence*, and *beliefs about consequences*. Cronbach’s alpha ranged from 0.65 (beliefs about consequences) to 0.92 (beliefs about capabilities). The values assigned to each item were added to create a composite score for each of the nine scales. A total of 11 items did not have a good fit in the scales for which they were initially intended; three fit in another scale, and the remaining eight did not fit in any scale and were analyzed as individual variables. Many significant correlations were found across the composite scores for each domain or sub-domain (supplementary file # 5).

### Definition of intention to offer PrEP care

We classified participants into different stages of intention according to the transtheoretical model of change [33]: unwilling (those who expressed that they were unlikely or very unlikely to offer PrEP care or have never considered it), pre-contemplation stage (those who reported being extremely likely or very likely to want to offer PrEP but likely, unlikely, or very unlikely to have the intention or a plan to do it), contemplation stage (extremely or very likely to have the intention but likely, unlikely, or very unlikely to have a plan), and planning stage (extremely or very likely to have a plan to offer PrEP care).

### Definition of advocating for PrEP

HCP were asked “how likely or unlikely would it be that you will advocate for PrEP in your clinic” and classified in three categories: extreme unlikely/very unlikely, likely, and very likely/extremely likely to advocate for PrEP.

### Additional measurements

Demographic questions cover aspects of gender (women vs. men), profession (physicians vs non-physicians), and years working in HIV care (less than 5 years, between 5 and 10, more than 10 years and less than 20, more than 20 years).

### Analysis

Nearly 92% of participants answered at least 70% of the questionnaire. During the data analysis, we found that a set of questions had been consistently left unanswered by some non-physician HCP. The reason for this was that certain activities inquired were outside the scope of practice for certain disciplines, e.g., a psychologist would not evaluate PrEP eligibility. As such, we did not treat these responses as missing data and carried out the analysis with participants providing complete data. A descriptive analysis was done for all survey items to explore data distribution and sample variability. Then, we assessed the association between the CFIR/TDF domains or subdomains and the intention to provide or advocate for PrEP. For this, we used two types of analyses. The first, confidence interval-based estimation (CIBER) analysis, was used to evaluate the univariate relationship between each of the items of each domain with the outcomes “intention” and “advocacy” [34]. The second, multinomial regressions, was performed to assess associations between each CFIR/TDF domain or subdomain and the outcome “intention,” and each CFIR/TDF domain or subdomain and the outcome “advocating,” while adjusting for confounders. To obtain a final multivariate model, we conducted a stepwise forward regression for each outcome: at each step, the variables that were significant at *P* level < 0.05 were included, and the variables at *p* > 20 were excluded. The best model for each outcome was confirmed using AIC and BIC criteria. Since participants were clustered in the HIV clinics and these clinics may have different structures, facilities, intentions, and experiences, we considered the 16 HIV clinics as clusters in the regression models. The multinomial regressions were done using STATA’s clustered sandwich estimator [35].

## Results

### Demographics

Of the 338 HCP invited to participate, 140 started the survey; and 100 had complete data in all variables studied and were included in the analyses. The demographic distribution of participants and the distribution of each of the CFIR/TDF items used in the survey is presented in Tables 1 and 2, and 3 of supplementary file # 6.

### CIBER analysis

Overall, HCP scored on the right side, meaning more positive perceptions or attitudes (supplementary file #7). Most items of *intervention characteristics*, *attitudes*, *professional role,* and *social influences* were related to one or both outcomes. Strong associations were also observed for specific items and the intention to offer PrEP care: those related to relative advantage in the *intervention characteristics*, using tools/scales to identify PrEP-eligible persons in the scale of *beliefs about capabilities*, and the statement “PrEP will be a very good fit in the clinic” in the *professional role*. The items within *concerns of the use of PrEP in populations* were related to the advocacy outcome. In the scale of *beliefs about consequences*, two items, namely worthiness and priority, were strongly related to both outcomes. Lastly, we observed that the items that did not load in any of the scales were correlated to the outcome of advocacy for PrEP.

### Multinomial analysis of “intention to offer PrEP care”

The adjusted predicted probabilities of intention after considering the clinics’ clusters were the following: 13.2% of HCPs were unwilling to offer PrEP care, 10.6% were in pre-contemplation, 18.5% were in contemplation, and 57% were in the planning stage. Compared to physicians, non-physicians were more likely to be in the unwilling (OR = 2.5, 95% CI [1.00–6.33]) and contemplation stages (OR = 2.8, 95% CI [1.3–6.0]) than in the planning stage. There were no differences between physicians and non-physicians in the pre-contemplation stage vs planning stage (OR = 2.8; 95% CI 0.82–9.45). Being a non-physician was related to lower scores in *PrEP characteristics*, *attitudes towards population’ needs*, *knowledge*, *beliefs about capabilities*, *social influence*, and *the professional role scales*. Being a non-physician was also related to higher scores on negative beliefs about consequences (data not shown). Age was not related to the stage of change. Women were more likely than men to be in the contemplation than in the planning stage (OR = 4.8, 95% CI [1.73–13.5]), with no difference between the other stages and the planning stage. The longer the experience in HIV care, the lower the likelihood of being in the unwilling stage (OR = 0.49, 95% CI [0.24–0.98]) and the higher the likelihood of being in the contemplation stage (OR = 1.79, 95% CI [1.09–2.93]).

Table 2 shows the multinomial regression of the bivariate association of each domain or subdomain scale with intention, adjusted by profession (physicians vs non-physicians), as this variable was the most important confounder. Compared to HCPs in the planning stage, those in the unwilling stage had fewer positive *attitudes toward population needs* (AOR = 0.79). Similarly, a one-unit increase in either the *professional role* or *social influence* scores was related to 22% and 11% lower odds of being in the unwilling stage, respectively, while a higher score on the scale of *beliefs about consequences* was related to higher odds of being in the unwilling stage (AOR = 1.26). Interestingly, higher levels of concern about the healthcare system were associated with a lower likelihood of being in the willing than in the planning stage (AOR = 0.88). Associations were also found for the items that did not load in other domains (Table 2). In general, it was observed that HCP in the unwilling stage as compared to those in the planning stage had more negative perceptions of PrEP, such as the risk of medicalization (AOR = 1.31), risk compensation (AOR = 1.78), ethics issues (AOR = 2.20), and misuse of resources (AOR = 1.71). Having control over the decision to offer PrEP was not related to the intention. HCPs who felt that taking care of people in PrEP would be the most important work in the clinic were less likely to be in the unwilling stage (AOR = 0.37). In the multivariate analysis (Table 3), four scales remained associated with the intention to offer PrEP care, preserving the same direction of the association: *attitudes towards patient needs*, *concerns of the health system*, *social norms*, and *beliefs about consequences*. None of the individual items were associated with the outcome in the multivariate analysis (data not shown).

**Table 2:** Bivariate multinomial logistic regression of CFRI/TDF scales on intention to offer PrEP care (adjusted by profession—physicians vs non-physicians)

Scale/item	OR; 95% CI Unwilling vs plan	OR; 95% CI Willing vs plan	OR; 95% CI Intention vs plan	Overall ***p***-value
***I. Scale of characteristics of PrEP***	0.83; 0.60–1.01	0.92; 0.78–1.08	1.02; 0.89–1.17	0.14
***II. Population needs and resources***				
*1. Scale of attitudes*	0.79; 0.63–0.79	0.93; 0.75–1.15	0.99; 0.87–1.13	*P* < 0.001
*2. Scale of concerns*	0.96; 0.84–1.10	0.97; 0.83–1.15	1.03; 0.90–1.19	0.90
*3. Beliefs about consequences*				
*I believe the use of PrEP will increase stigma in populations at risk*	1.33 (0.87–2.02)	0.70 (0.49–1.00)	0.77 (0.31–1.90)	0.26
*I believe it is unethical to prescribe antiretrovirals to HIV-negative people*	2.20 (1.71–2.84)	1.05 (0.69–1.61)	1.34 (0.95–1.88)	< 0.001
*PrEP will do more harm than good if not carefully implemented*	1.21 (1.08–1.37)	0.96 (0.63–1.45)	1.02 (0.72–1.45)	0.01
*PrEP would take resources that could be better used to improve access to antiretroviral medications*	1.71 (1.38–2.12)	0.71 (0.43–1.15)	1.04 (0.68–1.57)	< 0.001
*PrEP would lead to the use of medications for HIV prevention (medicalization of HIV prevention)*	1.31 (0.76–2.24)	0.85 (0.53–1.36)	0.77 (0.53–1.12)	0.06
*I believe PrEP would result in a reduction in condom use*	1.78 (1.08–2.92)	0.80 (0.50–1.29)	0.72 (0.38–1.36)	< 0.001
*I believe PrEP would increase to other sexually transmitted infections.*	1.28 (1.00–1.65)	0.85 (0.57–1.27)	0.81 (0.48–1.36)	< 0.001
**III. Characteristics of health system**				
*1. The Colombian healthcare system is not ready to support the implementation of PrEP*	0.80 (0.52–1.24)	1.26 (0.89–1.76)	0.97 (0.64–1.48)	0.31
2. Scale of Concerns	0.88; 0.81–0.95	0.99; 0.88–1.12	1.04; 0.95–1.14	0.002
***IV Characteristics of individuals***				
*1. Knowledge scale*	1.02; 0.95–1.09	0.95; 0.81;1.17	1.01; 0.93–1.09	0.72
*2. Scale of beliefs about capabilities*	0.97; 0.93–1.01	0.97; 0.92–1.00	1.00; 0.96;1.01	0.009
*3. Scale of social, professional role/compatibility*	0.78; 0.70–0.88	0.92; 0.73–1.18	0.97; 0.79–1.19	*P* < 0.001
*4. Social influence scale*	0.77; 0.64–0.93	0.98; 0.80–1.21	1.07; 1.00–1.015	< 0.001
*5. Scale of beliefs about consequences*	1.26; 1.15–1.41	0.96; 0.88–1.04	0.99; 0.80–1.22	< 0.001
*6. Control over the decision to offer PrEP care*	0.62 (0.35–1.09)	0.70 (0.51–0.96)	0.73 (0.47–1.11)	0.08
7..*Beliefs about consequences in HCP*				
Providing PrEP care would be the most important work I could do in the clinic	0.37 (0.225–0.53)	0.59 (0.30–1.13)	1.07 (0.80–1.44)	< 0.001
Providing PrEP care will be a good use of my time	0.63 (0.48–0.82)	0.70 (0.46–1.07)	1.04 (0.72–1.50)	< 0.001

**Table 3:** Multivariate multinomial logistic regression of CFIR/TDF scales on intention to offer PrEP care (adjusted by profession—physicians vs non-physicians)

Scale/item	OR; 95% CI Unwilling vs plan	OR; 95% CI Willing vs plan	OR; 95% CI Intention vs plan	Overall ***p***-value
***II. Population needs and resources***				
*1. Scale of attitudes*	0.68; 0.57–0.80	0.93; 0.75–1.13	0.99; 0.88–1.10	*P* < 0.001
**III. Characteristics of health system**				
2. Scale of concerns	0.88; 0.81–0.95	0.99; 0.88–1.12	1.04; 0.95–1.14	0.02
***IV Characteristics of individuals***				
*4. Social influence scale*	0.88; 0.74–1.04	0.99; 0.81–1.21	1.08; 1.01–1.16	< 0.001
*5. Scale of beliefs about consequences*	1.16; 1.03–1.30	0.93; 0.84–1.03	0.99; 0.83–1.18	0.002

### Associations with “advocating for PrEP in the clinic”

A total of 66.7% of the participants were likely and very likely to advocate for PrEP. Non-physicians were more likely to be unwilling to advocate for PrEP implementation in their clinics, 20.3%, than physicians, 6% (OR = 5.05; 95% CI 1.46–17.4). Neither age, sex, nor years of experience in HIV care were related to the intention to advocate for PrEP (data not shown). After adjusting for the profession of HCP (physicians vs non-physicians), higher scores in positive attitudes towards PrEP characteristics, and positive attitudes towards the needs of PrEP in populations were related to a lower likelihood of unwillingness to advocate (Table 4). Similarly, a higher level of concern on the use of PrEP in populations was related to higher odds of unwillingness to advocate. HCP with *higher beliefs about capabilities*, *higher scores in the professional role*, and *social influence scales* were less likely to be unwilling to advocate for PrEP. The more the negative attitudes in terms of the worthiness of PrEP and beliefs about consequences, the higher the likelihood of disfavoring PrEP implementation. All the individual items related to negative consequences of the use of PrEP in populations were associated with the likelihood of advocating for PrEP, whereas those endorsing more negative beliefs were more likely to disfavor the implementation of PrEP (Table 4). Those expressing control over the decision to offer PrEP care were less likely to be unwilling to advocate for PrEP. In the multivariate model, the scales of *intervention characteristics*, *attitudes towards patient needs*, and *concerns of use of PrEP in populations*, remained related to the outcome; of the individual items, five remained significant in the multivariate analysis, preserving the same direction (Table 5).

**Table 4:** Bivariate multinomial logistic regression of CFIR/TDF scales and individual items on advocate for PrEP care (adjusted by profession—physicians vs non-physicians)

Scale/item	OR; 95% CI Extreme unlikely/very unlikely vs very likely/extremely likely	OR; 95% CI Likely vs very likely/extremely likely	Overall ***p***-value
***I. Scale of characteristics of PrEP***	0.80; 0.69–0.92	0.82; 0.69–0.92	0.004
***II. Population needs and resources***			
*1. Scale of attitudes*	0.79; 0.63–0.79	0.93; 0.75–1.15	*P* < 0.001
*2. Scale of concerns*	1.10; 1.02–1.19	1.20; 1.09–1.32	< 0.001
*3. Beliefs about consequences*			
*I believe the use of PrEP will increase stigma in populations at risk*	1.97 (0.87–4.46)	1.83 (1.34–2.50)	< 0.001
*I believe it is unethical to prescribe antiretrovirals to HIV-negative people*	1.59 (0.83–3.03)	1.62 (1.12–2.33)	0.01
*PrEP will do more harm than good if not carefully implemented*	1.67 (1.08–2.60)	1.41 (1.02–1.96)	0.04
*PrEP would take resources that could be better used to improve access to antiretroviral medications*	1.71 (1.16–2.52)	1.74 (1.26–2.39)	< 0.001
*PrEP would lead to the use of medications for HIV prevention (medicalization of HIV prevention)*	2.88 (1.96–4.23)	1.14 (0.73–1.79)	0.06
*I believe PrEP would result in a reduction in condom use*	2.82 (1.54–5.13)	2.32 (1.47–3.67)	< 0.001
*I believe PrEP would result in an increase of other sexually transmitted infections.*	1.73 (1.20–2.49)	1.31 (0.81–2.10)	0.004
**III. Characteristics of health system**			
*1. The Colombian healthcare system is not ready to support the implementation of PrEP*	1.29 (0.78–2.13)	1.03 (0.58–1.83)	0.55
2. Scale of concerns	0.93; 0.84–1.03	0.96; 0.87–1.06	0.45
***IV Characteristics of individuals***			
*1. Knowledge scale*	0.94; 0.88–1.00	0.96; 0.86;1.06	0.14
*2. Scale of beliefs about capabilities*	0.96; 0.92–0.99	0.98; 0.95–1.01	0.01
*3. Scale of social, professional role/ compatibility*	0.78; 0.59–1.01	0.81; 0.69–0.94	0.02
*4. Social influence scale*	0.71; 0.59–0.87	0.69; 0.53–0.92	0.03
*5. Scale of beliefs about consequences*	1.18; 1.04–1.35	1.10; 0.93–1.30	0.03
*6. Control over the decision to offer PrEP care*	0.57 (0.38–0.85)	0.71 (0.51–0.99)	0.005
7.. *Beliefs about consequences in HCP*			
Providing PrEP care would be the most important work I could do in the clinic	0.57 (0.23–1.41)	0.72 (0.47–1.12)	0.21
Providing PrEP care will be a good use of my time	0.56 (0.40–0.80)	0.72 (0.53–0.97)	< 0.001

**Table 5:** Multivariate multinomial logistic regression of TDF and CFRI scales and individual items on advocate for PrEP care (adjusted by profession—physicians vs non-physicians)

Scale/item	OR; 95% CI Extreme unlikely/very unlikely vs very likely/extremely likely	OR; 95% CI Likely vs very likely/extremely likely	Overall ***p***-value
***I. Scale of characteristics of PrEP***	0.69; 0.52–0.92	0.86; 0.72–1.03	0.02
***II. Population needs and resources***			
*1. Scale of attitudes*	0.81; 0.68–0.96	0.85; 0.74–0.98	< 0.001
*2. Scale of concerns*	0.87; 0.73–1.02	1.08; 0.96–1.22	0.14
*3. Beliefs about consequences*			
*PrEP would take resources that could be better used to improve access to antiretroviral medications*	1.28 (0.62–2.64)	1.46 (1.12–1.92)	< 0.001
*PrEP would lead to the use of medications for HIV prevention (medicalization of HIV prevention)*	11.6 (3.59–37.9)	1.19 (0.54–2.62)	< 0.001
*I believe PrEP would result in a reduction condom use*	1.19 (0.49–2.86)	1.68 (1.12–2.05)	< 0.001
***IV Characteristics of Individuals***			
*6. Control over the decision to offer PrEP care*	0.42 (0.29–0.61)	0.76 (0.42–0.1.40)	< 0.001

## Discussion

This is one of the first studies to explore the determinants to PrEP implementation among HCP in Latin America. Our findings fill an important knowledge gap on PrEP with relevance for Colombia and possibly elsewhere. First, we developed and validated a questionnaire in the Spanish and English languages using the CFIR and TDF models to assess factors that could influence the stage of behavior change in HCP of HIV clinics regarding offering PrEP care or advocating for PrEP. We identified good construct validity and high reliability for most of the domains examined, and thus the scales and the items could be used to assess determinants of the intention of HCP and readiness for PrEP implementation in various settings. In the USA, similar CFIR-based scales have been found useful in the evaluation of the readiness of family planning clinics for PrEP implementation [10].

Secondly, we described the stage of change of HCP to offer PrEP care and the intention to advocate for PrEP. Consistent with other reports [20], non-physicians in this study were not only less likely to be in an advanced stage of adoption -they were less likely to want to offer PrEP care but also less likely to advocate for PrEP in their clinics. Non-physicians reported PrEP knowledge, perceptions, attitudes, beliefs, and concerns that may interfere with the effective delivery of PrEP care. Although non-physician HCP in Colombia cannot prescribe PrEP medications, they are expected to play instrumental roles in retention in care, education, and PrEP counseling as they already do in the clinics that care for people living with HIV. Others have highlighted the importance and need to include non-physician HCP in any initiative to improve PrEP delivery [36, 37]. These findings suggest that non-physicians will likely benefit from PrEP education to enhance their future contribution to PrEP delivery and avoid negative interference with key aspects of PrEP care. In contrast, HIV-experienced physicians and infectious disease specialists displayed strong intention to offer PrEP care, with 78% and 75% reporting being in the planning stage, respectively. Hence, HIV care-experienced physicians could also eventually function as PrEP champions in their clinics since promoting the visibility of successful adopters can accelerate adoption by other practitioners [38]. Importantly, several studies have reported that PrEP adoption and patient retention in care by primary care providers, HIV specialists, and other HCP remain low in Europe and North America (less than 30%) [39, 40], while a recent study in Brazil, reported that 70% of infectious disease doctors had not prescribed PrEP three years after its approval [41].

Lastly, the bivariate and multivariate models suggested that most of the CFIR/TDF domains or subdomains were related to the intention to either offer or advocate for PrEP. Perceptions of complexity, relative advantage, and the cost of PrEP set the conditions for PrEP implementation [8], with studies showing that more positive perceptions favor intentions as it was found in our sample regarding HCP advocacy [18]. In our sample, HCP show positive perceptions on most of the PrEP characteristics evaluated—efficacy, simplicity, and adaptability—except for relative advantage, where HCP show a degree of ambivalence. This ambivalence between using resources (medications) for treatment versus prevention could be related to the ethical issues raised by the use of medications for HIV-seronegative individuals which could compete for limited resources also needed for HIV care [14, 42].

Similar to the study of Piper et al. in HCP from family planning clinics in the USA [10], we have found that the HCP’s positive perceptions on the need for PrEP favors both outcomes, offering and advocating for PrEP. We have previously reported that HCP’s perception on the high need for PrEP in the population they serve was explained by their awareness of the nondecreasing incidence of HIV in Colombia, problems with antiretroviral drug adherence, and the lack of sexual health education [14]. Interestingly, HCP expressed experiencing low community pressure about PrEP use, as assessed by item seven (Table 1, supplementary file #3). This latter aspect could be explained by the low PrEP awareness in the populations at risk in Colombia [43, 44].

HCP were highly concerned about starting to use PrEP and about the readiness of the health system to support that intervention, with only the former concern being found to be related to advocacy. Other studies reported that healthcare system barriers impede PrEP prescription, even when options for coverage are available [11]. We found that concerns about PrEP use, especially risk compensation, were related to higher odds of being in the unwilling vs. the planning stage, a finding also reported by other studies [18, 20]. These concerns are legitimate while HCP anticipate PrEP implementation. An educational intervention that presents realistic expectations in terms of benefit/risk trade-offs in the context of PrEP will likely help ease such concerns.

Self-perceived PrEP knowledge was not an important determinant of intention to provide PrEP care, and it was related to advocacy in the bivariate analysis. We could not discriminate HCP by the level of knowledge since specific questions to assess PrEP knowledge were not asked. However, PrEP knowledge was correlated to *attitudes towards population needs and beliefs about capabilities*. The need for education on PrEP was more evident in nurses, pharmacists, and psychologists [45] who would eventually participate in PrEP provision via risk assessment, education, and counseling [46]. Notably, knowledge of PrEP has been found to influence PrEP prescription practices in other studies [47]. Early adopters of interventions are characterized by high levels of self-efficacy, and this may be the case of the physicians in this study, who were highly experienced in providing care to people living with HIV. Other studies have defined a set of knowledge and skills needed to effectively provide PrEP care, which could be used to develop training interventions and practice tools for HCP in Colombia [48]. Since educational interventions are feasible and effective for increasing HCP’s PrEP knowledge and confidence in its delivery [49, 50], tailored PrEP training interventions will need to be rolled out.

HCP who felt that PrEP was compatible with their practice, were more likely to have an intention to offer PrEP or advocate for PrEP, a finding consistent with a report of family planning clinics in the USA [10]. We explored this further in qualitative interviews and found that concerns on moral and religious values may hinder their willingness to offer PrEP [14]. Interpersonal stigma against MSM and TGW have been also described in other studies as factors hindering PrEP prescription [18]. One study in Brazil indicated that infectious disease doctors who declared a religion had more frequent concerns about risk compensation and lower willingness to prescribe PrEP to people who reported condomless sex [41]. Therefore, frank discussions about the importance of avoiding the HCP’s moral values affecting PrEP therapeutic decisions should be central in PrEP training. Additionally, emphasis should be made on normalizing education on HIV prevention to all population groups and not only to the traditionally high-risk groups, and training of HCP in anti-stigma practices and gender competence [51].

The last positive determinant found in our study was that related to *the social influence sub-domain***,** which consists of items related to social support and norms. In this study, the relationship between social norms and having a plan to offer PrEP care highlights the importance of PrEP prescription as a social practice, that is, HCP were more likely to offer PrEP care or advocate for PrEP if they have a positive working environment in which HCP felt encouraged to offer this service and colleagues have positive views of PrEP. Others have assessed this as a positive organizational climate for change [10], and it is consistent with the role of facilitating PrEP implementation and other evidence-based interventions [8, 52].

### Limitations and strengths

First, this study was conducted in the clinics of the VIHCOL network with a participation rate of 41%, which limits our ability to generalize our findings to HCP working in HIV clinics that did not participate in this study. Our study does not represent primary care providers whom would be eventually relevant for later stages of PrEP implementation. Of note, our sample consists of HCP who might well have the highest level of PrEP familiarity in Colombia and, therefore, with the highest intentions. In this scenario, it is conceivable that capacity building needs would be even greater for other HCP in Colombia especially for those in primary care. We also saw variations in response rate according to the clinic, as 50% of the sample belonged to one single health managing organization (Empresa Promotora de Salud [EPS] of the Colombian health system) that administers health services in several Colombia cities. This organization was also involved in a demonstration pilot study, which could have increased the intention to offer PrEP in our study. We adjusted for the clinical center to take into account organizational aspects of the clinic that could be related to intention and advocacy. Other aspects related to determinants at the clinical level, such as rural/urban, private/public provision, and the resources available in the clinic were aspects assessed via semi-structured interviews with clinic managers [14]. Our results are consistent with other reports in the literature on the factors that influence PrEP provision by HCP. Future work on PrEP implementation will focus on primary care providers who readily interact with people at high risk of HIV acquisition (e.g., sex workers, HIV-negative TG and MSM).

We limited the examination of the CFIR and TDF items to a limited number of experts. The inclusion of additional experts could have led to a reduction or the inclusion of other items. Besides, a larger sample size could have allowed us to test all items with all latent constructs tested together, and perform a confirmatory factor analysis or a more powerful tool to model typologies or subgroups according to intentions [10]. However, we employed a rigorous methodology: we used robust implementation theoretical frameworks and standard practices to develop the survey, used best practices for data collection, included input from clinical PrEP experts, and performed sound analyses. Our questionnaire could be further improved in wording and content, be adapted to other settings or a different target sample.

## Conclusions

In this paper, we have described the currenct status of HCP’s intentions to offer/advocate for PrEP in the VIHCOL network of clinics. To generate sound implementation strategies, we will (1) triangulate this study’s findings with qualitative studies in people at risk of HIV, who are the potential future PrEP users, to identify needs that HCP can address through the practice of PrEP and, (2) delineate, by using intervention mapping, the most appropriate implementation strategies that fit the Colombian context [53]. Consistent with the CFIR/TDF model, our findings suggest a multilevel approach to increase the intention to offer PrEP or advocate for PrEP in HCP. First, there is a need to develop a training program for HCP that (1) teach skills that favor PrEP adoption [45, 48], (2) increase HCP’s knowledge of the population they serve including risk factors and PrEP barriers, (3) address main concerns about the use of PrEP, (4) include discussions about HCP’s moral values affecting PrEP therapeutic decisions, and (5) include anti-stigma practices and gender competence skills. Second, there is a need to support the role of more skilled professionals as PrEP champions since they can promote PrEP adoption by less experienced HCP, could model PrEP practices in their clinics, and become advocates of PrEP in their communities. Potential PrEP champions may benefit from leadership training to enhance their abilities to motivate change and effectively advocate for their communities. Lastly, structural approaches that focus on increasing resources for PrEP delivery, developing plans to cover the cost of PrEP-related services, and incorporating health care service navigators will likely promote PrEP adoption (5). There is a need to discuss this approach with managers and HCP at higher levels of leadership, and examine its effectiveness and cost.

## Supplementary Information

**Additional file 1.** Details of the Colombian health system.

**Additional file 2 **STROBE Statement—Checklist of items that should be included in reports of *cross-sectional studies*.

**Additional file 3: Table 1s.** Mapping CFIR domains, TDF and survey items.

**Additional file 4.** Full questionnaire in Spanish.

**Additional file 5.** Validity of the CFIR/TDF items.

**Additional file 6: Table 1s.** General characteristics of the full sample of participants. **Table 2s.** Distribution of CFIR and TDF items in the sample of HIV- HCP in Colombia. **Table 3s.** Distribution of CFIR and TDF items that did not fit in the scales- sample of HIV- HCP in Colombia.

**Additional file 7.** CIBER analysis.

## Data Availability

The datasets generated and/or analyzed during the current study are not publicly available but are available from the corresponding author on reasonable request. Data from this study could only be shared with the approval of the ethics committee of each institution. Researchers are welcome to contact BEA or HM for any request.

## Abbreviations

PrEP: HIV pre-exposure prophylaxis
TDF: Theoretical domains framework
CFIR: Consolidated framework for implementation research
HCP: Health care professionals
MSM: Men who have sex with men
TGW: Transgender women
EPS: Entidades promotoras de salud
