# Supplementary material for: Applying implementation science frameworks to identify factors that influence the intention of healthcare providers to offer PrEP care and advocate for PrEP in HIV clinics in Colombia: a cross-sectional study
Source: Implement Sci Commun. 2022 Mar 16;3:31. doi: 10.1186/s43058-022-00278-2 (PMC8925047; doi:10.1186/s43058-022-00278-2)
Supplement: Supplementary file 1 — Additional file 1. Details of the Colombian health system. [file 43058_2022_278_MOESM1_ESM.docx]

**Details of the Colombian health system**

In Colombia, *Entidades Promotoras de Salud* (EPS) affiliate individuals and manage health care resources under plans of care. Actual healthcare services are delivered by Instituciones Prestadoras de [Servicios de] Salud (IPS), namely clinics, medical centers, and hospitals that are contractually bound by one or more EPS to deliver the services needed by individuals and delineated by each EPS’s healthcare plans. IPS offer services to populations of various socioeconomic backgrounds that can vary according to geographical location (e.g. large city vs. small village) and availability of human resources and infrastructure. EPS pay IPS for services provided to affiliated individuals. The cost of health care services is funded by contributions from people with employment and medium to high-income levels (*regimen contributivo*). These funds also cover health services for people with low or no income (*regimen subsidiado*). Affiliation to the *regimen contributivo* is compulsory for employed individuals in Colombia. Healthcare services covered by these two regimens are delineated in the *Plan Obligatorio de Salud* (POS). Very high-income individuals can purchase additional healthcare services for a fee via private insurers (*medicina prepagada*).
