# Supplementary material for: Applying implementation science frameworks to identify factors that influence the intention of healthcare providers to offer PrEP care and advocate for PrEP in HIV clinics in Colombia: a cross-sectional study
Source: Implement Sci Commun. 2022 Mar 16;3:31. doi: 10.1186/s43058-022-00278-2 (PMC8925047; doi:10.1186/s43058-022-00278-2)
Supplement: Supplementary file 3 — Additional file 3: Table 1s. Mapping CFIR domains, TDF and survey items. [file 43058_2022_278_MOESM3_ESM.docx]

**Table 1s. Mapping CFIR domains, TDF and survey items**

| **CFIR DOMAINS** | **Survey items** | **Question, answers, and codification** |
| --- | --- | --- |
| **I. INTERVENTION CHARACTERISTICS** |  | **Total=10 items** |
| **A. Evidence Strength & Quality** | HCPs’ perceptions of the quality and validity of evidence support the belief that the intervention will have desired outcomes. |  |
|  | 1. PrEP would prevent HIV acquisition | How much disagree or agree with the following statements  Likert scale five options  Range:-2 (completely disagree)/2 (completely agree) |
|  | *2. I believe there are better strategies for HIV prevention than PrEP* |  |
|  | 3. We should start using PrEP as a prevention strategy for HIV as soon as possible |  |
| **B Relative advantage** | HCPs’ perception of the advantage of implementing the intervention versus an alternative solution |  |
|  | *1.PrEP would not be better than prevention programs already in place* | How much disagree or agree with the following statements  Likert scale five options  Range:-2 (completely disagree)/2 (completely agree) |
|  | 2. I believe PrEP would very well complement programs addressing condom use |  |
|  | 3. I believe PrEP would very well complement programs addressing the sexual health of populations at risk |  |
| **C Adaptability** | The degree to which an intervention can be adapted, tailored, refined, or reinvented to meet local needs.  1. It is possible to adapt PrEP protocols to the needs of populations at risk of HIV | How much disagree or agree with the following statements  Likert scale five options  Range:-2 (completely disagree)/2 (completely agree) |
| **D Complexity** | Perceived difficulty of implementation, reflected by duration, scope, radicalness, disruptiveness, centrality, and intricacy and number of steps required to implement.  1.PrEP is easy to implement | How much disagree or agree with the following statements  Likert scale five options  Range:-2 (completely disagree)/2 (completely agree) |
| **E Cost** | Costs of the intervention and costs associated with implementing that intervention include investment, supply, and opportunity costs. |  |
|  | 1. I think PrEP will be cheaper than HIV treatment | How much disagree or agree with the following statements  Likert scale five options  Range:-2 (completely disagree)/2 (completely agree) |
|  | *2. PrEP would take resources that could be better used to improve access to antiretroviral medications* |  |

*Note: Items in italic means negative perceptions about PrEP.*

| **CFIR DOMAINS** | **Survey items** | **Question, answers, and codification** |
| --- | --- | --- |
| **II. OUTER SETTING** |  | Total=20 items |
| **A. Patient Needs & Resources** | The extent to which people’s need for PrEP is recognized by HCP, and barriers and facilitators to meet those needs. This domain includes aspects such as attitudes of HCP towards the need for PrEP (negative and positive), HCP’s perceptions of the consequences of PrEP use in the population, and HCP concerns regarding the use of medications for PrEP and condoms. |  |
| **A1. Attitudes towards the need for PrEP in populations** | *1. In Colombia, there are very few people in need of using PrEP* | How much disagree or agree with the following statements  Likert scale five options  Range:-2 (completely disagree)/2 (completely agree) |
|  | *2. If PrEP is implemented in the clinic it would not be welcomed by the patients* |  |
|  | *3. I do not believe that the population at risk of HIV is interested in using PrEP as a prevention strategy* |  |
|  | *4. I believe it is unethical to prescribe antiretrovirals to HIV-negative people* |  |
|  | 5. PrEP is something that people who receive care in this clinic want |  |
|  | 6. PrEP is something people at risk of HIV want |  |
|  | 7. There is adequate support from LGTBI community organizations for PrEP implementation |  |
|  | 8. PrEP would have positive effects in populations at risk of HIV |  |
| **A2. Concerns about the use of PrEP in populations** | *1. Emergence of drug resistance* | How much of a concern are the following aspects related to PrEP  Likert scale five options  Range: 1 (not a concern)/ 5 ( lot of concern) |
|  | *2. Toxicity of the medications in people who are HIV negative* |  |
|  | *3. That adherence to medications ends up being poor* |  |
|  | *4. That persons on PrEP poorly engage with the monitoring visits* |  |
|  | *5. That people on PrEP lower their condom use.* |  |
|  | *6. Lack of evidence on its effectiveness for HIV prevention* |  |
|  | *7. Inappropriate use of PrEP medication- illegally selling them, counterfeiting them.* |  |
| **A3. Beliefs about consequences of use of PrEP in populations** | *1. I believe PrEP would result in a reduction in condom use* | How much disagree or agree with the following statements  Likert scale five options  Range:-2 (completely disagree)/2(completely agree) |
|  | *2. I believe the use of PrEP will increase stigma in populations at risk* |  |
|  | *3. PrEP would lead to the use of medications for HIV prevention (medicalization of HIV prevention)* |  |
|  | *4. PrEP will do more harm than good if not carefully implemented* |  |
|  | *5. I believe PrEP would increase to other sexually transmitted infections.* |  |
| *Note: Items in italic means negative attitudes or beliefs about PrEP* | | |
| **CFIR DOMAINS** | **Survey items** | **Question, answers, and codification** |
| **III. HEALTH SYSTEMS** |  | **Total=6** |
| **A. Attitudes** | Attitudes of HCP regarding the preparedness of the health system to implement PrEP. |  |
|  | *1. The Colombian healthcare system is not ready to support the implementation of PrEP* | How much disagree or agree with the following statements  Likert scale five options; where -2 (completely disagree)/2(completely agree |
| **B. Concerns** | Concerns of HCP regarding the preparedness of the health system to implement PrEP |  |
|  | 1. *That the healthcare system does not approve the medications used for PrEP* | How much of a concern are the following aspects related to PrEP  Likert scale five options where 1 (not a concern)/ 5 (a lot of concern) |
|  | 1. *That health plans do not include PrEP in their prevention protocols* |  |
|  | 1. *That the healthcare system does not allow to cover the follow-up visits of people on PrEP* |  |
|  | 1. *That the time needed for monitoring and counseling people taking PrEP is not allocated* |  |
|  | *5. That the healthcare personnel is not adequately trained in the care of PrEP patients* |  |

*Note: Items in italic means negative attitudes or beliefs about PrEP*

| **CFIR DOMAINS** | **Survey items** | **Question, answers, and codification** |  |
| --- | --- | --- | --- |
| **IV. CHARACTERISTICS OF INDIVIDUALS** |  | Total=29 items |  |
| **A Knowledge** | HCP are aware of PrEP as an HIV prevention strategy and are familiar with the delivery of PrEP components |  |  |
|  | 1. Efficacy of PrEP | From 1 to 5; where 1 is no knowledge at all to 5 where is good knowledge, how you classify your knowledge of PrEP  Numerical scale |  |
|  | 2. Frequency and severity of side effects |  |  |
|  | 3. Identification of people who could benefit |  |  |
|  | 4. Counselling people for PrEP |  |  |
|  | 5. Medications for use in PrEP |  |  |
| **B Beliefs about capabilities** /self-efficacy | The self-confidence of HCP in performing activities related to PrEP and implementing PrEP |  |  |
|  | 1.can effectively offer PrEP care | On a scale from 1 to 10, where 1 is not at all and 10 totally confident how confident are you that you…  Numerical scale |  |
|  | 2. can provide counseling to people on PrEP |  |  |
|  | 3. can effectively use the algorithms to identify people for PrEP |  |  |
|  | 4. can offer PrEP care if I have a clear protocol at hand |  |  |
|  | 5 . can collaborate effectively with colleagues in offering PrEP in my clinic |  |  |
| **C Professional role/compatibility** | The extent to which PrEP implementation will be/is perceived by HCP as part of their work or responsibilities, or **compatible with their work**. |  |  |
|  | 1.I see my values reflected in the implementation of PrEP | How much disagree or agree with the following statements  Likert scale five options; where -2 (completely disagree)/2(completely agree |  |
|  | 2.Providing PrEP care will be compatible with my work in the clinic |  |  |
|  | 3. I see the values of the clinic reflected in the implementation of PrEP |  |  |
|  | 4. PrEP will be a very good fit in my clinic |  |  |
|  | *5. PrEP should not be implemented in the clinic* |  |  |
| **D Social influences** | Peer opinions about PrEP may influence the implementation of PrEP. This includes aspects related to social norms (people think they should do PrEP), group norm (people are motivated and they think will support PrEP) |  |  |
| **D1. Social norms** | 1.Many of my colleagues will approve that I offer PrEP care | How much disagree or agree with the following statements  Likert scale five options; where -2 (completely disagree)/2(completely agree |  |
|  | 2.Many colleagues think that it will be important for me to offer PrEP care |  |  |
| **D2 Group norms** | 1.In the clinic there are many people motivated to offer PrEP care |  |  |
|  | 2.I think my colleagues would support the implementation of PrEP in the clinic |  |  |
| **E. Control** | HCP perceptions that they have control over the decision to offer PrEP care | How much disagree or agree with the following statements  Likert scale five options; where -2 (completely disagree)/2(completely agree |  |
|  | 1. The decision to offer PrEP care would depend on me |  |  |
| **F Individual Stage of Change** | HCP intentions to offer PrEP care or advocate for PrEP in the clinic |  |  |
|  | 1. Want to offer PrEP care | If in the next 12 months tenofovir and emtricitabine become available in Colombia for PrEP, how likely or unlikely is that you  From 1 to 5, where 1 is very likely, and 5 very unlikely |  |
|  | 2. Have the intention to offer PrEP care |  |  |
|  | 3. Have a plan to offer PrEP care |  |  |
|  | 4. I will advocate for the implementation of PrEP in the Clinic |  |  |
| **G. Beliefs about consequences** | It refers to HCP beliefs about the value of PrEP if they manage people in PrEP (offer PrEP care), if they expect a worthy outcome – in terms of time- if there are consequences, rewards or incentives for managing people in PrEP. |  |  |
| **G1. Worthiness** | *1. Providing PrEP care is not worth it* | If in the future, PrEP is implemented in your clinic, How much do you agree (or disagree) with the following statements  Likert scale five options; where -2 (completely disagree)/2(completely agree |  |
|  | 2. Providing PrEP care would be the most important work I could do in the clinic |  |  |
|  | 3. Providing PrEP care will be a good use of my time |  |  |
| **G2. Outcomes** | *4. Providing PrEP care will require more time than I have* |  |  |
| **G3. Priority** | *5. Providing PrEP care would not be a priority to me* |  |  |
| **G4. Reinforcement** | 1. If I offer PrEP care, I would obtain a financial benefit |  |  |
|  | 2. If I offer PrEP care I will receive recognition from professionals who are important to me |  |  |

*Note: Items in italic means negative attitudes or beliefs about PrEP*
