# Supplementary material for: Applying implementation science frameworks to identify factors that influence the intention of healthcare providers to offer PrEP care and advocate for PrEP in HIV clinics in Colombia: a cross-sectional study
Source: Implement Sci Commun. 2022 Mar 16;3:31. doi: 10.1186/s43058-022-00278-2 (PMC8925047; doi:10.1186/s43058-022-00278-2)
Supplement: Supplementary file 4 — Additional file 4. Full questionnaire in Spanish. [file 43058_2022_278_MOESM4_ESM.docx]

DESARROLLO DE ESTRATEGIAS DE IMPLEMENTACIÓN de PrEP EN CLÍNICAS DEL VIH EN COLOMBIA

End of Block: CONSENTIMIENTO INFORMADO

Start of Block: FAMILIARIDAD

| 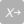 |
| --- |

Como describiría su nivel de conocimiento sobre la profilaxis pre-exposición para el VIH (PrEP)?

- Nunca he oído de PrEP
- Conozco muy poco de PrEP
- Conozco algo PrEP
- Conozco de PrEP
- Conozco mucho de PrEP

Ubíquese en la barra y marque de 1 a 5, qué tanto conoce usted las guías de atención de personas en PrEP  (En donde 1 es no las conozco y 5 es las conozco a profundidad)

|  | 1 | 2 | 3 | 4 | 5 |
| --- | --- | --- | --- | --- | --- |

| Guías del CDC |  |
| --- | --- |
| Guías de PrEP de la Organización Mundial de la Salud |  |
| Guías Latinoamericanas |  |

| Page Break |  |
| --- | --- |

Ubíquese en la barra y marque de 1 a 5, qué tanto conoce  usted sobre  los siguientes aspectos  En donde es 1 no se nada y 5 es tengo conocimiento profundo)

|  | 1 | 2 | 3 | 4 | 5 |
| --- | --- | --- | --- | --- | --- |

| Eficacia de PrEP en la prevención del VIH |  |
| --- | --- |
| Frecuencia y severidad de los efectos adversos de medicamentos de PrEP |  |
| Identificación de personas que podrían beneficiarse de tomar PrEP |  |
| Consejería relacionada con PrEP |  |
| Medicamentos que se usan para PrEP |  |

| 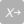 | 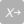 |
| --- | --- |

Dónde ha oído o leído información sobre PrEP? <

- Revistas científicas
- Conferencias
- Colegas
- Talleres de capacitación
- Pacientes
- Sitios de internet
- Periodicos
- Otros ________________________________________________

 Ha usted leído algún otro recurso o material para informarse sobre PrEP?

- Si, cual? ________________________________________________
- No

| 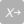 |
| --- |

La informacióndisponible sobre PrEP es a su parecer:

- 1- Fácil de encontrar
- 2
- 3
- 4
- 5- Díficil de encontrar

| 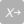 |
| --- |

La información disponible sobre PrEP es a su parecer:

- 1 -Fácil de entender
- 2
- 3
- 4
- 5- Díficil de entender

| Page Break |  |
| --- | --- |

End of Block: FAMILIARIDAD

Start of Block: Definición de PrEP

Breve recuento sobre que es PrEP

De acuerdo con la Organización Mundial de la Salud (OMS),  se define a la PrEP como el uso de una medicación antirretroviral en personas no infectadas por VIH con el fin de prevenir que ellas adquieran la infección por VIH La PrEP se puede tomar por vía oral, utilizando un fármaco antirretroviral disponible para el tratamiento de la infección por VIH (tenofovir más emtricitabina) o tópicamente como un gel vaginal que contiene tenofovir. La eficacia de la PrEP oral se ha demostrado en varios ensayos controlados aleatorios y es alta cuando el fármaco se utiliza según las indicaciones. La recomendación es tomar los medicamentos de forma diaria, continua y sin interrupciones durante el tiempo que se continúe bajo prácticas sexuales de riesgo. Existen evidencias científicas sobre su eficacia para prevenir la adquisición de infección por VIH. La PrEP no protege de otras infecciones de transmisión sexual, los embarazos y tampoco son una cura para la infección por el VIH.  PrEP no es lo mismo que la profilaxis posterior a la exposición o PEP, que es el uso diario de píldoras antirretrovirales durante 28 días DESPUÉS de una posible exposición. Hasta el momento los medicamentos antiretrovirales para uso en PrEP no han sido aprobados en Colombia.

< Las siguientes preguntas se relacionan con las opiniones que las personas tienen sobre PrEP. Por favor marque la respuesta que más se acerca a lo que Usted piensa

| 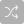 | 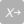 |
| --- | --- |

Que tanto esta Ud de acuerdo o en desacuerdo con las siguientes afirmaciones

|  | Completamente de acuerdo | De acuerdo | Ni de acuerdo ni en desacuerdo | En desacuerdo | Completamente en desacuerdo | No sé |
| --- | --- | --- | --- | --- | --- | --- |
| PrEP podría hacer más daño que bien, si no se implementa con cuidado |  |  |  |  |  |  |
| En Colombia hay muy poca gente que necesita usar PrEP |  |  |  |  |  |  |
| Creo que el uso de PrEP conllevaría a menor uso del condón |  |  |  |  |  |  |
| Creo que no es ético dar medicamentos antirretrovirales a personas que no tienen el VIH |  |  |  |  |  |  |
| Creo que el uso de PrEP conduciría al incremento de otras enfermedades de transmisión sexual |  |  |  |  |  |  |

| 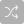 | 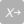 |
| --- | --- |

Que tanto esta Ud de acuerdo o en desacuerdo con las siguientes afirmaciones

|  | Completamente de acuerdo | De acuerdo | Ni de acuerdo ni en desacuerdo | En desacuerdo | Completamente en desacuerdo | No sé |
| --- | --- | --- | --- | --- | --- | --- |
| PrEP puede prevenir la adquisición de VIH |  |  |  |  |  |  |
| PrEP debería empezar a usarse tan pronto como sea posible como estrategia de prevención del VIH |  |  |  |  |  |  |
| Creo que PrEP complementaría muy bien los programas de promoción de uso de condón |  |  |  |  |  |  |
| El uso de PrEP tendría efectos positivos en las personas a riesgo de VIH |  |  |  |  |  |  |
| Pienso que PrEP sería más barato que el tratamiento de VIH |  |  |  |  |  |  |
| Los servicios de salud de Colombia no estan listos para apoyar el uso de PrEP |  |  |  |  |  |  |
| PrEP conllevaría al uso de medicamentos en la prevención de VIH (medicalización de la prevención) |  |  |  |  |  |  |

| 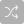 | 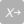 |
| --- | --- |

Que tanto esta Ud de acuerdo o en desacuerdo con las siguientes afirmaciones

|  | Completamente de acuerdo | De acuerdo | Ni de acuerdo ni en desacuerdo | En desacuerdo | Completamente en desacuerdo | No sé |
| --- | --- | --- | --- | --- | --- | --- |
| Creo que el uso de PrEP incrementaría el estigma en las poblaciones de riesgo |  |  |  |  |  |  |
| Creo que hay mejores alternativas para la prevención de VIH que PrEP |  |  |  |  |  |  |
| Creo que PrEP es un buen complemento para mejorar la salud sexual de las poblaciones a riesgo |  |  |  |  |  |  |
| PrEP no sería mejor que los programas de prevención ya existentes |  |  |  |  |  |  |
| PrEP tomaría recursos que podrían usarse para mejorar el acceso a los antirretrovirales |  |  |  |  |  |  |

| Page Break |  |
| --- | --- |

| 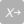 |
| --- |

< Con respecto a PrEP, que tanto le preocuparía

|  | 1- Nada | 2- Poco | 3- Algo | 4-Bastante | 5 -Mucho |
| --- | --- | --- | --- | --- | --- |
| La toxicidad de los medicamentos de PrEP en personas sin VIH |  |  |  |  |  |
| Falta de evidencia de su efectividad respecto a la prevención de VIH |  |  |  |  |  |
| Aparición de resistencia a medicamentos |  |  |  |  |  |
| Personal no capacitado en manejo de personas en PrEP |  |  |  |  |  |
| Que la adherencia a los medicamentos no sea óptima |  |  |  |  |  |
| El mal uso de los medicamentos de PrEP, e.j. venta ilegal, adulteración |  |  |  |  |  |

| Page Break |  |
| --- | --- |

| 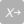 |
| --- |

Con respecto a PrEP, que tanto le preocuparía

|  | 1- Nada | 2- Poco | 3- Algo | 4-Bastante | 5 -Mucho |
| --- | --- | --- | --- | --- | --- |
| Que las personas no cumplan con las citas de monitoreo de PrEP |  |  |  |  |  |
| Menor uso de condón en personas en PrEP |  |  |  |  |  |
| Que el sistema de salud no apruebe los medicamentos para ser usados en PrEP |  |  |  |  |  |
| Falta de tiempo para monitorear y aconsejar a las personas que toman PrEP |  |  |  |  |  |
| Que los planes de beneficio de salud no incluyan PrEP en sus protocolos de prevención |  |  |  |  |  |
| Que el sistema de salud no permita dar citas de seguimiento a personas en PrEP |  |  |  |  |  |

| Page Break |  |
| --- | --- |

Tiene alguna preocupación sobre PrEP que pudiera comentar?

________________________________________________________________

________________________________________________________________

________________________________________________________________

________________________________________________________________

________________________________________________________________

End of Block: ACTITUDES

Start of Block: Definicion de adminitrador

¿En qué entidad de salud ejerce regularmente su práctica clínica o asistencial en VIH? El nombre de la entidad no aparecerá en ninguna publicación ni reporte. A las clínicas les será asignado un número de identificación.

________________________________________________________________

| 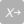 |
| --- |

Cuantos años de trabajo tiene en esta clínica?

- Menos de 5 años
- De 5 a 10 años
- Más de 10 años- Menos de 15 años
- Más de 15 años

| Page Break |  |
| --- | --- |

| 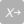 |
| --- |

Cuantos pacientes con VIH atiende a la semana?

- De 1 a 24
- De 25 a 49
- De 50 a 74
- Más de 75
- No atiendo pacientes

| 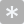 |
| --- |

Qué porcentaje de su tiempo en la clínica participa usted en las siguientes actividades (la sumatoria de las actividades debe ser igual a 100%)

_______ Atención de pacientes

_______ Administración

_______ Investigación

_______ Entrenamiento de profesionales

_______ Dirección

| 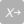 |
| --- |

| 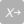 |
| --- |

<p>La clínica tendrá un plan claro para implementar PrEP </p>

- Extremadamente probable
- Muy probable
- Probablemente
- Poco probable
- Nada probable

|  |
| --- |

<

End of Block: POBLACIONES A LAS QUE SIRVE LA CLINICA Y SU PERCEPCION

Start of Block: PERFIL PROFESIONAL Y PREFERENCIAS

Para finalizar, algunas preguntas sobre Usted

| 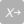 |
| --- |

Su profesión o especialidad es:

- Médico(a) general
- Médico(a) especialista no infectologo
- Médico experto en VIH
- Medico Infectólogo
- Enfermera
- Químico farmacéutico
- Trabajador Social
- Psicólogo
- Otro ________________________________________________

| Page Break |  |
| --- | --- |

| 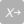 |
| --- |

Cuántos años de trabajo profesional en VIH tiene?

- Menos de 5 años
- Más de 5 menos de 10 años
- Más de 10 años menos de 20 años
- Más de 20 años

| Page Break |  |
| --- | --- |

End of Block: PERFIL PROFESIONAL Y PREFERENCIAS

Start of Block: EXPERIENCIA Y AUTO-EFICACIA

| Page Break |  |
| --- | --- |

| 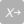 |
| --- |

Alguna vez ha recibido entrenamiento, capacitación, o tomado un curso específico en:

|  | Si | No |
| --- | --- | --- |
| Guías de manejo de personas en PrEP |  |  |
| Atención de personas en PrEP |  |  |
| Consejería de personas en PrEP |  |  |
| Uso de instrumentos para identificar personas para PrEP |  |  |

| Page Break |  |
| --- | --- |

| 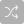 | 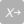 |
| --- | --- |

En que medida esta Ud de acuerdo con la siguiente afirmación:

|  | Totalmente de acuerdo | De acuerdo | Ni de acuerdo ni en desacuerdo | En desacuerdo | Totalmente en desacuerdo |
| --- | --- | --- | --- | --- | --- |
| Necesito más capacitación para atender personas en PrEP |  |  |  |  |  |

Las siguientes preguntas se refieren a algunas capacidades que Ud podría necesitar para la implementación de PrEP. Trate de responder cada afirmación y sólo use la opción de - NO relevante-cuando no aplique a su práctica clínica En una escala de 1 a 10, donde 1 es para nada seguro y 10 totalmente seguro, <span style="font-size:16px;"> que tan seguro(a) esta Usted de que </div>

|  | No relevante |
| --- | --- |

|  | 1 | 2 | 3 | 4 | 5 | 6 | 7 | 8 | 9 | 10 |
| --- | --- | --- | --- | --- | --- | --- | --- | --- | --- | --- |

| Realmente puedo atender personas en PrEP | 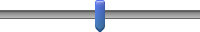 |
| --- | --- |
| Puedo brindar consejería a personas en PrEP | 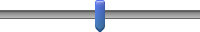 |
| Realmente puedo usar los algoritmos de identificación de personas para PrEP | 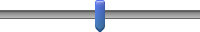 |
| Puedo atender personas en PrEP si tengo a la mano un protocolo claro de PrEP | 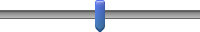 |
| Puedo colaborar efectivamente con otros colegas para usar PrEP en mi práctica clínica | 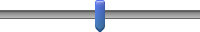 |

| Page Break |  |
| --- | --- |

| 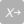 |
| --- |

¿Alguna vez alguien le ha preguntado sobre PrEP?

- Si
- No

| 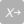 |
| --- |

<p>¿Cuántas veces le ha ocurrido eso en el último año?<o:p></o:p></p>

- Varias veces (al menos una vez a la semana)
- Algunas veces (al menos una vez al mes)
- Muy rara vez (menos de 10 veces al año)
- Casi nunca (1 o 2 veces al año)

| 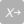 |
| --- |

¿Quién le preguntó de PrEP? -<br> <em>Marque todas las que apliquen</em>

- Gay/HSH
- Bisexual
- Transgénero: hombre a mujer
- Transgénero: mujer a hombre
- Usadores de drogas
- Trabajadores sexuales
- Personas VIH-negativa que están en una relación de pareja o tiene un compañero/a seropositivo/a.
- Heterosexuales (hombre o mujer)
- Otro ________________________________________________

| Page Break |  |
| --- | --- |

| 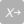 |
| --- |

<p>Ha Ud iniciado una conversación con alguno de sus pacientes acerca de PrEP?<o:p></o:p></p>

- Si
- No

| 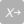 |
| --- |

¿Cuántas veces lo ha hecho en el último año?

- Varias veces (al menos una vez a la semana)
- Algunas veces (al menos una vez al mes)
- Muy rara vez (menos de 10 veces al año)
- Casi nunca (1 o 2 veces al año)

| 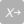 |
| --- |

Con quien ha iniciado conversaciones acerca de PrEP-  Marque todas las que apliquen

- Gay/HSH
- Bisexual
- Transgénero: hombre a mujer
- Transgénero: mujer a hombre
- Usadores de drogas
- Trabajadores sexuales
- Personas VIH-negativa que están en una relación de pareja o tiene un compañero/a seropositivo/a.
- Heterosexuales (hombre o mujer)
- Otro ________________________________________________

| Page Break |  |
| --- | --- |

| 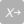 |
| --- |

Cuando habla de PrEP, usted se siente...

- Entusiasta
- Neutral
- No entusiasmado/a
- Nervioso/a
- Nunca he hablado

| Page Break |  |
| --- | --- |

| 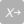 |
| --- |

Alguna vez ha atendido personas en PrEP?

- Si
- No

| 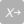 |
| --- |

Cuantas personas ha atendido en PrEP en el último año?

- Menos de 5
- Entre 5 y 10
- Más de 10

| Page Break |  |
| --- | --- |

| 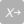 |
| --- |

A que grupos de población ha manejado en PrEP?

- Gay/HSH
- Bisexual
- Transgénero: hombre a mujer
- Transgénero: mujer a hombre
- Usadores de drogas
- Trabajadores sexuales
- Personas VIH-negativa que están en una relación de pareja o tiene un compañero/a seropositivo/a.
- Heterosexuales (hombre o mujer)
- Otro ________________________________________________

| Page Break |  |
| --- | --- |

| 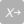 |
| --- |

Con que frecuencia en el pasado ha formulado tratamiento post-exposición para el VIH- PEP- o ha manejado personas en PEP ?

- No aplica, este trabajo no lo hago
- Varias veces (al menos una vez a la semana)
- Algunas veces (al menos una vez al mes)
- Muy rara vez (menos de 10 veces al año)
- Casi nunca (1 o 2 veces al año)

Las siguientes preguntas se relaciona con su experiencia y comodidad con algunas practicas clínicas relacionadas con la atención de personas que viven con VIH o la atención a personas que están a riesgo de VIH

| Page Break |  |
| --- | --- |

| 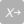 |
| --- |

A cuantas de las personas que usted atiende en la clínica:<o:p></o:p></p>

|  | Todos | La mayoría de ellos | Alguno de ellos | A muy pocos | A ninguno |
| --- | --- | --- | --- | --- | --- |
| Les pregunta su historia sexual (e.j. numero, edad y genero de las parejas, edad de inicio de relaciones sexuales, abuso sexual, antecedentes de enfermedades de transmisión sexual) |  |  |  |  |  |
| Les evalua comportamientos de riesgo o preventivos (e.j. uso de condón, sexo comercial, uso de drogas, abuso de alcohol) |  |  |  |  |  |

| Page Break |  |
| --- | --- |

| 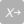 |
| --- |

  A cuantas de las personas que usted atiende en la clínica:

|  | Todos | La mayoría de ellos | Alguno de ellos | A muy pocos | A ninguno |
| --- | --- | --- | --- | --- | --- |
| Les pregunta la orientación sexual |  |  |  |  |  |
| Les pregunta sobre la seropositividad al VIH de los compañeros sexuales |  |  |  |  |  |
| Les proporciona consejería para reducción de riesgo de VIH |  |  |  |  |  |
| Les pregunta su identidad de género |  |  |  |  |  |

| 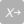 |
| --- |

 Con que frecuencia usted :<o:p></o:p></p>

|  | Nunca | Varias veces en el año | Una vez al mes | Varias veces en el mes | Una vez a la semana | Varias veces a la semana | Todos los días |
| --- | --- | --- | --- | --- | --- | --- | --- |
| Ofrece una prueba para diagnóstico de VIH |  |  |  |  |  |  |  |
| Hace consejería en adherencia a medicamentos para el VIH |  |  |  |  |  |  |  |
| Informa a una persona el diagnóstico de VIH |  |  |  |  |  |  |  |
| Pregunta sobre efectos adversos de medicamentos para el VIH |  |  |  |  |  |  |  |
| Hace monitoreo de medicamentos para el VIH |  |  |  |  |  |  |  |
| Ofrece pruebas de diagnóstico de otras enfermedades de transmisión sexual |  |  |  |  |  |  |  |

| Page Break |  |
| --- | --- |

| 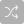 | 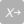 |
| --- | --- |

Como describiría su nivel de comodidad ?<o:p></o:p></p>

|  | Totalmente cómodo(a) | Muy cómodo (a) | Más o menos cómodo (a) | Poco cómodo(a) | Para nada cómodo(a) | No aplica |
| --- | --- | --- | --- | --- | --- | --- |
| Indagando sobre la historia sexual de un paciente (e.j. numero, edad y genero de las parejas, edad de inicio de relaciones sexuales, abuso sexual, antecedentes de enfermedades de transmisión sexual) |  |  |  |  |  |  |
| Evaluando comportamientos de riesgo o preventivos (e.j. uso de condón, sexo comercial, uso de drogas, abuso de alcohol) |  |  |  |  |  |  |

| Page Break |  |
| --- | --- |

| 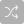 | 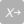 |
| --- | --- |

Como describiría su nivel de comodidad ?

|  | Totalmente cómodo(a) | Muy cómodo (a) | Más o menos cómodo (a) | Poco cómodo(a) | Para nada cómodo(a) | No aplica |
| --- | --- | --- | --- | --- | --- | --- |
| Preguntando la orientación sexual de un paciente |  |  |  |  |  |  |
| Preguntando sobre la seropositividad al VIH de los compañeros sexuales |  |  |  |  |  |  |
| Proporcionando consejería para reducción de riesgo de VIH |  |  |  |  |  |  |
| Preguntando la identidad de género de un paciente |  |  |  |  |  |  |

| Page Break |  |
| --- | --- |

| 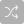 | 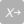 |
| --- | --- |

Como describiría su nivel de comodidad?

|  | Totalmente cómodo(a) | Muy cómodo (a) | Más o menos cómodo (a) | Poco cómodo(a) | Para nada cómodo(a) | No aplica |
| --- | --- | --- | --- | --- | --- | --- |
| Ofreciendo una prueba para diagnóstico de VIH |  |  |  |  |  |  |
| Haciendo consejería en adherencia de medicamentos para VIH |  |  |  |  |  |  |
| Informándole a una persona el diagnóstico de VIH |  |  |  |  |  |  |
| Manejando medicamentos para el VIH |  |  |  |  |  |  |
| Preguntando sobre efectos adversos de medicamentos para el VIH |  |  |  |  |  |  |
| Ofreciendo pruebas de diagnóstico de enfermedades de transmisión sexual |  |  |  |  |  |  |

| Page Break |  |
| --- | --- |

End of Block: EXPERIENCIA Y AUTO-EFICACIA

Start of Block: INTENCIONES DE USO DE PREP

Las siguientes preguntas se refieren a las intenciones personales para atender personas en PrEP.  se llegara a aprobar en Colombia el uso de tenofovir y emtricitabina para propósito de PrEP,  que tan probable o improbable es que Usted

| 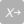 |
| --- |

Quiera atender personas en PrEP?

- Extremedamante probable
- Muy probable
- Probablemente
- Poco probable
- Nada probable
- No lo he considerado

| Page Break |  |
| --- | --- |

| 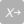 |
| --- |

Tenga un plan para atender personas en PrEP?

- Extremadamente probable
- Muy probable
- Probablemente
- Poco Probable
- Nada probable
- No lo he considerado

| Page Break |  |
| --- | --- |

| 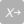 |
| --- |

Tenga la intención de atender personas en PrEP?</div>

- Extremadamente probable
- Muy probable
- Probable
- Poco probable
- Nada probable
- No lo he considerado

| Page Break |  |
| --- | --- |

| 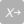 |
| --- |

Este dispuesto(a) a abogar por la implementación de PrEP en su institución?

- Extremadamente probable
- Muy probable
- Probable
- Poco probable
- Nada probable
- No lo he considerado

| Page Break |  |
| --- | --- |

| 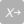 |
| --- |

La decisión de atender personas en PrEP dependerá completamente de mí

- Completamente de acuerdo
- De acuerdo
- Ni de acuerdo ni en desacuerdo
- En desacuerdo
- Totalmente en desacuerdo

| Page Break |  |
| --- | --- |

Qué factores podrían  facilitar  su intención de atender personas en PrEP ,En donde 1 es el que más podría facilitar y 6 el que menos  facilitaría.

______ Mas tiempo en la clínica para discutir riesgos y hacer monitoreo

______ Que las EPS autoricen los medicamentos

______ Que haya personas interesadas en tomar PrEP

______ Que haya guías nacionales apoyando el manejo de personas en PrEP

______ Más entrenamiento en PrEP

______ Más estudios demostrando eficacia

Habría algún otro facilitador que no este incluido en la pregunta de arriba?

________________________________________________________________

________________________________________________________________

________________________________________________________________

________________________________________________________________

________________________________________________________________

| Page Break |  |
| --- | --- |

En orden de importancia,  quiénes podrían influenciar en Ud la intención de atender personas en PrEPEn donde 1 es quien más podría influenciar y 6 el que menos pudiera influenciar

______ Colegas de mi clínica

______ Administrativos de mi clínica

______ Directivos de EPS

______ Expertos en PrEP nacionales

______ Ministerio de Salud

______ Expertos en PrEP internacionales

Otra persona u organización que podría influenciar esta decisión?

________________________________________________________________

< Si en un futuro se llegara a implementar la provisión de servicios de  PrEP en su clínica/Institución, qué tanto estaría usted de acuerdo o en desacuerdo con las siguientes afirmaciones</p>

|  | Totalmente de acuerdo | De acuerdo | Ni de acuerdo ni en desacuerdo | En desacuerdo | Totalmente en desacuerdo |
| --- | --- | --- | --- | --- | --- |
| Atender personas en PrEP no sería una prioridad para mí |  |  |  |  |  |
| La atención de personas en PrEP no valdría la pena |  |  |  |  |  |
| Atender personas en PrEP requerirá más tiempo del que tengo en la clínica |  |  |  |  |  |
| Si atendiera personas en PrEP obtendría un beneficio financiero |  |  |  |  |  |
| Atender personas en PrEP será un uso valioso de mi tiempo |  |  |  |  |  |
| Atender personas en PrEP sería el trabajo más importante que podría hacer en la clínica |  |  |  |  |  |

| Page Break |  |
| --- | --- |

| 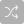 | 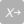 |
| --- | --- |

< Si en un futuro se llegará a implementar la provisión de servicios de  PrEP en su clínica/Institución, qué tanto estaría usted de acuerdo o en desacuerdo con las siguientes afirmaciones</p>

|  | Totalmente de acuerdo | De acuerdo | Ni de acuerdo ni en desacuerdo | En desacuerdo | Totalmente en desacuerdo | No sé |
| --- | --- | --- | --- | --- | --- | --- |
| Veo mis valores personales reflejados en la implementación de PrEP |  |  |  |  |  |  |
| Veo los valores de la clínica reflejados en la implementación de PrEP |  |  |  |  |  |  |
| Atender personas en PrEP sería compatible con mi trabajo en la clínica |  |  |  |  |  |  |
| PrEP encajaría bien en mi institución |  |  |  |  |  |  |
| PrEP es simple de implementar en la clínica |  |  |  |  |  |  |
| PrEP no debería implementarse en la clínica |  |  |  |  |  |  |

End of Block: INTENCIONES DE USO DE PREP

Start of Block: REDES E INFLUENCIA

| 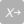 |
| --- |

Con respecto a PrEP, que tanto esta Usted de acuerdo o en desacuerdo con las siguientes afirmaciones

|  | Totalmente de acuerdo | De acuerdo | Ni de acuerdo ni en desacuerdo | En desacuerdo | Totalmente en desacuerdo | No sé |
| --- | --- | --- | --- | --- | --- | --- |
| Muchos colegas a mi alrededor aprobarían que yo atendiera personas en PrEP |  |  |  |  |  |  |
| Por atender personas en PrEP recibiría reconocimiento de profesionales que son importantes para mí |  |  |  |  |  |  |
| Muchos colegas piensan que sería importante que atendiera personas en PrEP |  |  |  |  |  |  |
| En la clínica hay bastantes personas motivadas en la atención de personas en PrEP |  |  |  |  |  |  |
| Creo que mis colegas apoyarían la implementación de PrEP en la clínica |  |  |  |  |  |  |
| Muchos colegas a mi alrededor desaprueban el uso de PrEP |  |  |  |  |  |  |

| Page Break |  |
| --- | --- |

End of Block: REDES E INFLUENCIA

Start of Block: OR4kt

Las siguientes preguntas se enfocan en aspectos organizacionales de la clínica o institución donde usted presta atención a personas que viven con VIH. Estas preguntas son muy importantes para conocer factores que podrían facilitar la implementación de PrEP, y por ello es muy importante que conozcamos sus opiniones sobre ellos.  Le recordamos que esta información sobre la clínica es anónima, y no será compartida con colegas ni directores sino como datos agregados.  Para contestar este cuestionario debe pensar en un cambio, intervención, o implementación de nuevas prácticas que hayan realizado en la clínica y que estén relacionadas con la atención de pacientes o prevención del VIH. <

| Page Break |  |
| --- | --- |

| 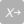 |
| --- |

En su clínica

|  | Totalmente de acuerdo | En acuerdo | Ni de acuerdo ni en desacuerdo | En desacuerdo | Totalmente en desacuerdo |
| --- | --- | --- | --- | --- | --- |
| Existe voluntad para ajustarse a los cambios |  |  |  |  |  |
| Existe capacidad para intercambiar ideas y tener impacto sobre las decisiones vinculadas a la atención del paciente |  |  |  |  |  |
| Existe la flexibilidad necesaria para ajustarse al cambio |  |  |  |  |  |
| Los profesionales están dispuestos a hacer ajustes en el trabajo habitual en respuesta a lo que sucede a su alrededor |  |  |  |  |  |
| Generalmente, se pueden adaptar nuevas normas o procedimientos al trabajo habitual, incluso los que son impuestos |  |  |  |  |  |

En su clínica

|  | Totalmente de acuerdo | En acuerdo | Ni de acuerdo ni en desacuerdo | En desacuerdo | Totalmente en desacuerdo |
| --- | --- | --- | --- | --- | --- |
| Existen experiencias previas de implementación de prácticas clínicas o preventivas, obtenida de proyectos o programas pilotos o sus evaluaciones |  |  |  |  |  |
| Los directivos promueven el cambio comportándose de manera cosistente con dicho cambio en las prácticas clínicas o preventivas |  |  |  |  |  |
| Los directivos definen el curso del cambio en las prácticas clínicas o preventivas |  |  |  |  |  |
| Se dispone de conocimientos sobre cómo están siendo utilizadas por otras organizaciones innovaciones similares en las prácticas clínicas o preventivas |  |  |  |  |  |
| Los directivos conocen la innovación gracias a su experiencia previa |  |  |  |  |  |

| 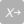 |
| --- |

En su clínica

|  | Totalmente de acuerdo | En acuerdo | Ni de acuerdo ni en desacuerdo | En desacuerdo | Totalmente en desacuerdo |
| --- | --- | --- | --- | --- | --- |
| Los cambios propuestos han sido bien recibidos por los pacientes |  |  |  |  |  |
| Los cambios propuestos toman en consideración las necesidades y preferencias de los pacientes |  |  |  |  |  |
| Los cambios propuestos parecen tener más ventajas que desventajas para los pacientes |  |  |  |  |  |
| Los cambios propuestos deberían ser efectivos, basados en los conocimientos científicos actuales |  |  |  |  |  |

| Page Break |  |
| --- | --- |

| 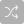 | 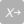 |
| --- | --- |

En su clínica

|  | Totalmente de acuerdo | En acuerdo | Ni de acuerdo ni en desacuerdo | En desacuerdo | Totalmente en desacuerdo |
| --- | --- | --- | --- | --- | --- |
| Los procesos de cambio son monitorizados continuamente |  |  |  |  |  |
| Los resultados son monitorizados continuamente |  |  |  |  |  |
| La evaluación y la mejora de la implementación de cambio incluye un plan de divulgación de resultados |  |  |  |  |  |
| La evaluación y la mejora de la implementación de cambio incluye la revisión de resultados por los líderes |  |  |  |  |  |
| Hay mecanismos formales establecidos para recoger los comentarios relacionados con el cambio propuesto |  |  |  |  |  |

| Page Break |  |
| --- | --- |

| 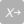 |
| --- |

En su clínica

|  | Totalmente de acuerdo | En acuerdo | Ni de acuerdo ni en desacuerdo | En desacuerdo | Totalmente en desacuerdo |
| --- | --- | --- | --- | --- | --- |
| Los profesionales trabajan en equipo |  |  |  |  |  |
| Los profesionales están habitualmente pendientes de ayudarse entre sí cuando se necesita |  |  |  |  |  |
| La confianza mutua entre profesionales es fuerte |  |  |  |  |  |
| La sobrecarga de trabajo reduce la efectividad de las intervenciones |  |  |  |  |  |
| La frustación de los profesionales es frecuente |  |  |  |  |  |

| Page Break |  |
| --- | --- |

| 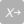 |
| --- |

En su clínica

|  | Totalmente de acuerdo | En acuerdo | Ni de acuerdo ni en desacuerdo | En desacuerdo | Totalmente en desacuerdo |
| --- | --- | --- | --- | --- | --- |
| Las ideas y sugerencias de los profesionales reciben la debida consideración por parte de la dirección |  |  |  |  |  |
| los canales de comunicación formales funcionan muy bien |  |  |  |  |  |
| Los profesionales se sienten libres para formular preguntas y expresar sus preocupaciones |  |  |  |  |  |
| La dirección esta abierta a las ideas de los profesionales para propiciar los cambios |  |  |  |  |  |
| La dirección favorece prácticas nuevas y diferentes |  |  |  |  |  |

| Page Break |  |
| --- | --- |

| 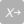 |
| --- |

En su clínica, los profesionales

|  | Totalmente de acuerdo | De acuerdo | Neutral | En desacuerdo | Totalmente en desacuerdo |
| --- | --- | --- | --- | --- | --- |
| Tienen un sentido de responsabilidad para mejorar la atención del paciente y sus resultados |  |  |  |  |  |
| Cooperan para mantener y mejorar la efectividad de la atención del paciente |  |  |  |  |  |
| Están dispuestos a innovar o experimentar para mejorar los procedimientos clínicos |  |  |  |  |  |
| Son receptivos al cambio en los procesos clínicos |  |  |  |  |  |

| Page Break |  |
| --- | --- |

| 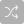 | 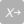 |
| --- | --- |

En su clínica

|  | Totalmente de acuerdo | En acuerdo | Ni de acuerdo ni en desacuerdo | En desacuerdo | Totalmente en desacuerdo |
| --- | --- | --- | --- | --- | --- |
| Los pacientes presionan para que se realicen cambios en las prácticas clínicas o preventivas |  |  |  |  |  |
| Las presiones para realizar cambios en las prácticas clínicas o preventivas provienen de los profesionales |  |  |  |  |  |
| La dirección presiona para realizar cambios en las prácticas clínicas o preventivas |  |  |  |  |  |
| Las presiones para realizar cambios en las prácticas clínicas o preventivas provienen de las EPS |  |  |  |  |  |
| Las secretarias de salud presionan para realizar cambios en las prácticas clínicas o preventivas |  |  |  |  |  |

| 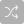 | 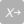 |
| --- | --- |

En su clínica

|  | Totalmente de acuerdo | En acuerdo | Ni de acuerdo ni en desacuerdo | En desacuerdo | Totalmente en desacuerdo |
| --- | --- | --- | --- | --- | --- |
| Los miembros del equipo brindan apoyo real a las nuevas ideas y sus aplicaciones |  |  |  |  |  |
| Los miembros del equipo ponen fácil el desarrollo de nuevas ideas |  |  |  |  |  |
| Los miembros del equipo cooperan para ayudar a desarrollar y aplicar nuevas ideas |  |  |  |  |  |
| Entre los miembros del equipo se comparten recursos para facilitar la aplicación de nuevas ideas |  |  |  |  |  |

| Page Break |  |
| --- | --- |

| 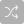 | 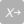 |
| --- | --- |

En su clínica

|  | Totalmente de acuerdo | En acuerdo | Ni de acuerdo ni en desacuerdo | En desacuerdo | Totalmente en desacuerdo |
| --- | --- | --- | --- | --- | --- |
| La dirección proporciona una gestión eficaz para la mejorar continua de la atención al paciente |  |  |  |  |  |
| La dirección ofrece a los profesionales retroalimentación- datos sobre los efectos de las decisiones clínicas |  |  |  |  |  |
| La dirección pide cuenta a los empleados de los resultados alcanzados |  |  |  |  |  |
| La dirección participa en los procesos de cambio |  |  |  |  |  |
| Los profesionales participan en los procesos de cambio |  |  |  |  |  |
| El personal no clínico participa en los procesos de cambio |  |  |  |  |  |

En la clínica

|  | Totalmente de acuerdo | En acuerdo | Ni de acuerdo ni en desacuerdo | En desacuerdo | Totalmente en desacuerdo |
| --- | --- | --- | --- | --- | --- |
| Los agentes externos participan en el proceso de planificacion |  |  |  |  |  |
| Generalmente, todos los empleados están involucrados en los procesos de toma de decisiones |  |  |  |  |  |
| Hay un responsable de las decisiones de innovación entre los clínicos |  |  |  |  |  |
| Hay un responsable de las decisiones de innovación entre los administrativos |  |  |  |  |  |
| La dirección recompensa la innovación clínica y la creatividad para mejorar la atención del paciente |  |  |  |  |  |
| La dirección busca la forma de mejorar la educación del paciente y aumentar su participación en el tratamiento |  |  |  |  |  |

| 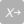 |
| --- |

La clínica

|  | Totalmente de acuerdo | En acuerdo | Ni de acuerdo ni en desacuerdo | En desacuerdo | Totalmente en desacuerdo |
| --- | --- | --- | --- | --- | --- |
| Tiene capacidad de determinar roles y responsabilidades para implementar nuevas prácticas clínicas o de prevención |  |  |  |  |  |
| Tiene el apoyo necesario en términos de presupuesto o recursos económicos para implementar nuevas prácticas clínicas o de prevención |  |  |  |  |  |
| Tiene el apoyo necesario en términos de formación para nuevas prácticas clínicas o de prevención |  |  |  |  |  |
| Tiene el apoyo necesario en término de instalaciones y equipos para nuevas prácticas clínicas o de prevención |  |  |  |  |  |
| Tiene el apoyo necesario en cuanto a la dotación de personal para nuevas prácticas clínicas o de prevención |  |  |  |  |  |

End of Block: OR4kt

Start of Block: Final de la encuesta

Muchas gracias por su participación, la información que nos ha dado será muy importante para definir pautas de implementación de PrEP en Colombia.

End of Block: Final de la encuesta
