# Supplementary material for: Applying implementation science frameworks to identify factors that influence the intention of healthcare providers to offer PrEP care and advocate for PrEP in HIV clinics in Colombia: a cross-sectional study
Source: Implement Sci Commun. 2022 Mar 16;3:31. doi: 10.1186/s43058-022-00278-2 (PMC8925047; doi:10.1186/s43058-022-00278-2)
Supplement: Supplementary file 5 — Additional file 5. Validity of the CFIR/TDF items. [file 43058_2022_278_MOESM5_ESM.doc]

**Validity of the CFIR/TDF items**

**Results of exploratory factor analysis with polychoric correlations. The final models and internal coherence are presented. HCP of HIV clinics in Colombia**

**I. Characteristics of PrEP**

| **Items and domains** | **Alpha=0.83** |
| --- | --- |
| We should start using PrEP a prevention strategy for HIV as soon as possible | 0.80 |
| I believe PrEP would very well complement programs addressing condom use | 0.80 |
| *I believe there are better strategies for HIV prevention than PrEP.* | 0.60 |
| I believe PrEP would very well complement programs addressing the sexual health of populations at risk | 0.72 |
| *PrEP would not be better than prevention programs already in place* | 0.47 |
| I think PrEP will be cheaper than HIV treatment | 0.67 |
| PrEP would prevent HIV adquisition. | 0.57 |

**II. Outter settings- Population needs and resources**

| **Items and domains** | **Factor loadings** |
| --- | --- |
| ***A1. Attitudes towards population needs/willingness*** | **Alpha= 0.70** |
| *In Colombia, there are very few people in need of using PrEP* | -0.51 |
| *I do not believe that the population at risk of HIV is interested in using PrEP as a prevention strategy* | -0.50 |
| *If PrEP is implemented in the clinic it would not be welcome by the patients* | -0.43 |
| *I believe it is unethical to prescribe antiretrovirals to HIV-negative people* | -0.63 |
| PrEP is something that people who receive care in this clinic want | 0.46 |
| PrEP is something people at risk of HIV want. | 0.67 |
| There is adequate support from LGTBI community organizations for PrEP implementation | 0.45 |
| PrEP would have positive effects in populations at risk of HIV | 0.61 |
| It is possible to adapt PrEP protocols to the needs of populations at risk of HIV* | 0.60 |
| ***A2. Concerns in people who will use PrEP*** | **Alpha=0.83** |
| *Toxicity of the medications in people who are HIV negative* | 0.56 |
| *Emergence of drug resistance* | 0.69 |
| *That adherece to medications ends up being poor* | 0.89 |
| *That persons on PrEP poorly engage with the monitoring visits* | 0.80 |
| *That people on PrEP lower their condom use.* | 0.71 |
| *Inappropriate use PrEP medication- illegally selling them, counterfeiting them.* | 0.81 |

**III. Health Systems**

| ***B. Concerns about health systems*** | **Alpha =0.85** |
| --- | --- |
| *That the healthcare system does not approve the medications used for PrEP* | 0.80 |
| *That health plans do not include PrEP in their prevention protocols* | 0.85 |
| *That the healthcare system do not allow to cover the follow-up vistis of people on PrEP* | 0.86 |
| *That the time needed for monitoring and counseling people taking PrEP is not allocated* | 0.63 |
| *That the healthcare personnel is not adequately trained in the care of PrEP patients* | 0.50 |

**IV. Individual characteristics**

| ***A. Knowledge- familiarity*** | **Alpha=0.92** |
| --- | --- |
| Efficacy of PrEP | 0.89 |
| Frequency and severity of side effects | 0.85 |
| Identification of people who could benefit | 0.85 |
| Counselling people for PrEP | 0.83 |
| Medications for use in PrEP | 0.75 |
| ***B. Beliefs about capability*** | **Alpha=*0.92*** |
| … can effectively offer PrEP care | 0.95 |
| … can provide counseling to people on PrEP | 0.94 |
| … can effectively use the algorithms to identify people for PrEP | 0.75 |
| … can offer PrEP care if I have a clear protocol at hand | 0.68 |
| … can collaborate effectively with colleagues in offering PrEP in my clinic | 0.83 |
| ***D. Social influences*** | **Alpha=0.77** |
| Many of my colleagues will approve that I offer PrEP care | 0.65 |
| Many colleagues think that it will be important for me to offer PrEP care | 0.77 |
| In the clinic there are many people motivated to offer PrEP care | 0.72 |
| I think my colleagues would support the implementation of PrEP in the clinic | 0.69 |
| If I offer PrEP care I will receive recognition of professionals who are important to me* | 0.63 |
| **C. Social/professional role** | **Alpha=0.80** |
| I see my personal values reflected in the implementation of PrEP | 0.66 |
| Providing PrEP care will be compatible with my work in the clinic | 0.79 |
| I see the values of the clinic reflected in the implementation of PrEP | 0.78 |
| PrEP will be a very good fit in my clinic | 0.84 |
| PrEP is easy to implement* | 0.61 |
| **G. Beliefs about consequences** | **Alpha=0.65** |
| *Providing PrEP care would not be a priority to me* | 0.76 |
| *Providing PreP care is not worth it* | 0.77 |
| *Providing PrEP care will require more time than I have* | 0.40 |
| If I offer PrEP care, I would obtain a financial benefit | 0.59 |

* Items that load in other domains that the one it was initially tested.

**Correlations (Bonferroni Adjusted)**

Many significant correlations were found across the composited scores for each domain or sub-domain (data not shown). First, we found positive attitudes on PrEP characteristics were related to positive attitudes towards population needs (0.30; p=0.01) and negatively with beliefs about consequences (*r* = -0.32; p=0.01). The scores of attitudes towards population needs were correlated with higher scores in knowledge (*r* = 0.32, p=0.05), higher scores in social influence (*r* = 0.32, p=0.02), higher professional roles (*r* = 0.40, p=0.0003), and negative correlated with beliefs about consequences in HCP (*r* = -0.37, p=0.001). A high correlation was present between population and system concerns (*r* = 0.62; p<0.001), but no other correlations were found between the concern variables and the other variables. The scale of knowledge was related to higher scores in beliefs about capabilities (*r* = 0.47; p<0.001). Last, HCPs with higher scores in social influence also had higher scores in the professional role scale (*r* = 0.34; p=0.007).
