# Supplementary material for: Applying implementation science frameworks to identify factors that influence the intention of healthcare providers to offer PrEP care and advocate for PrEP in HIV clinics in Colombia: a cross-sectional study
Source: Implement Sci Commun. 2022 Mar 16;3:31. doi: 10.1186/s43058-022-00278-2 (PMC8925047; doi:10.1186/s43058-022-00278-2)
Supplement: Supplementary file 6 — Additional file 6: Table 1s. General characteristics of the full sample of participants. Table 2s. Distribution of CFIR and TDF items in the sample of HIV- HCP in Colombia. Table 3s. Distribution of CFIR and TDF items that did not fit in the scales- sample of HIV- HCP in Colombia. [file 43058_2022_278_MOESM6_ESM.docx]

**Table 1s. General characteristics of the full sample of participants.**

|  | **Total** | |
| --- | --- | --- |
| **Age (average; SD)** | **37.5; 9.3** |  |
|  | **n** | **%** |
| **Sex** |  |  |
| Male | **51** | **39.5** |
| Female | **78** | **60.5** |
| **Years of HIV work** |  |  |
| Less than 5 years | **59** | **48.4** |
| 5 - 10 years | **32** | **26.2** |
| More than 10 years | **31** | **25.1** |
| **Health Discipline** |  |  |
| General Physician | **12** | **9.3** |
| Specialist-(other than Infectious Disease specialist) | **3** | **2.3** |
| Physician HIV specialist | **34** | **26.4** |
| Infectious Disease specialist | **6** | **4.6** |
| Nurse | **31** | **24.0** |
| Pharmacist | **14** | **10.8** |
| Social worker | **9** | **6.9** |
| Psychologist | **16** | **12.4** |
| Other | **4** | **3.1** |

**Table 2s. Distribution of CFIR and TDF items in the sample of HIV- HCP in Colombia.**

| **I. INTERVENTION CHARACTERISTICS** | **Survey items** | **Mean, SD** |  |
| --- | --- | --- | --- |
|  | 1. PrEP would prevent HIV acquisition | 1.31;0.78 | 90.7% |
|  | *2. I believe there are better strategies for HIV prevention than PrEP* | 0.04; 1.06 | 35.4% |
|  | 3. We should start using PrEP as a prevention strategy for HIV as soon as possible | 0.95; 1.06 | 70.5% |
|  | *4.PrEP would not be better than prevention programs already in place* | 0.36;1.03 | 46.5% |
|  | 5. I believe PrEP would very well complement programs addressing condom use | 0.96;0.96 | 75.0% |
|  | 6. I believe PrEP would very well complement programs addressing the sexual health of populations at risk | 0.75;1.06 | 71.3% |
|  | 7. I think PrEP will be cheaper than HIV treatment | 0.89; 1.04 | 70.5% |
| **Total scale** | Higher scores =more positive attitudes in regards to advantage, efficacy, positive effects | 4.51;3.21(-4/14) |  |

*Note: Items in italic means negative perceptions about PrEP.*

**Table 2s. Distribution of CFIR and TDF items in the sample of HIV- HCP in Colombia**

| **CFIR DOMAINS** | **Survey items** |  |  |
| --- | --- | --- | --- |
| **A1. Attitudes** |  | **Mean; SD** | **% agree and completely agree** |
|  | *1. In Colombia, there are very few people in need of using PrEP* | -1.34; 0.81 | 3.88% |
|  | *2. If PrEP is implemented in the clinic it would not be welcomed by the patients* | -0.77(1.10) | 13.18% |
|  | *3. I do not believe that the population at risk of HIV is interested in using PrEP as a prevention strategy* | -1.14;0.96 | 10.86% |
|  | *4. I believe it is unethical to prescribe antiretrovirals to HIV-negative people* | -0.99; 1.12 | 11.8% |
|  | 5. PrEP is something that people who receive care in this clinic want | 0.40;0.91 | 46.51% |
|  | 6. PrEP is something people at risk of HIV want | 1.06;0.74 | 88.0% |
|  | 7. There is adequate support from LGTBI community organizations for PrEP implementation | 0.35;0.95 | 40.0% |
|  | 8. PrEP would have positive effects in populations at risk of HIV | 1.17; 0.84 | 86.0% |
|  | 9. It is possible to adapt PrEP protocols to the needs of populations at risk of HIV | 1.17;0.69 |  |
| **Total scale** | Higher scores =more positive attitudes towards the need for PrEP in populations, the possibility of adapting PrEP and the interest of LGTB communities | 8.25; 4.44; (-4;18) |  |
| **A2. Concerns about the use of PrEP in populations** |  | **Mean, SD** | **% who answered “a lot and extremely concern”** |
|  | *1. Emergence of drug resistance* | 3.6;1.15 | *22.9%* |
|  | *2. Toxicity of the medications in people who are HIV negative* | 3.30;1.09 | *43.7%* |
|  | *3. That adherence to medications ends up being poor* | 4.2;0.91 | *81.4%* |
|  | *4. That persons on PrEP poorly engage with the monitoring visits* | 4.2;0.83 | *83.7%* |
|  | *5. That people on PrEP lower their condom use.* | 4.3;0.90 | *82.8%* |
|  | *7. Inappropriate use of PrEP medication- illegally selling them, counterfeiting them.* | 4.40;0.82 | *86%* |
| Total scale | Higher scores = higher levels of concerns in regards to the use of PrEP in populations | 24;SD:4.4 (11-30) |  |
| **III. HEALTH SYSTEMS** | **Survey items** |  |  |
| **B. Concerns** |  | **Mean, SD** | **% a lot and extremely concern** |
|  | *1. That the healthcare system does not approve the medications used for PrEP* | 3.90; 1.07 | 68.7% |
|  | *2.That health plans do not include PrEP in their prevention protocols* | 3.87;0.98 | 65.6% |
|  | *3.That the healthcare system does not allow to cover the follow-up visits of people on PrEP* | 4.05;1.02 | 73.4% |
|  | *4.That the time needed for monitoring and counseling people taking PrEP is not allocated* | 3.81;1.09 | 67.9% |
|  | *5. That the healthcare personnel is not adequately trained in the care of PrEP patients* | 4.08;0.92 | 75.9% |
| Total scale | Higher scores= higher level of concerns in regards to health system coverage, financing and readiness | 19.5; 4.56; | Range: (1-25) |

*Note: Items in italic means negative attitudes or beliefs about PrEP*

**Table 2s. Distribution of CFIR and TDF items in the sample of HIV- HCP in Colombia**

| **CFIR DOMAINS** | **Survey items** |  |  |
| --- | --- | --- | --- |
| **IV. CHARACTERISTICS OF INDIVIDUALS** |  |  |  |
| **A Knowledge** |  | **Mean, SD** | **% Score 4 or 5** |
|  | 1. Efficacy of PrEP | 3.52;1.27 | 56.8% |
|  | 2. Frequency and severity of side effects | 3.36;1.33 | 52.3% |
|  | 3. Identification of people who could benefit | 3.85;1.19 | 68.2% |
|  | 4. Counselling people for PrEP | 3.36;1.34 | 47.7% |
|  | 5. Medications for use in PrEP | 3.87;1.30 | 62.1% |
| **Total scale** | Higher scores = more knowledge of clinical aspects of PrEP | 17.95;5.63;(5/25) |  |
| **B Beliefs about capabilities** |  | **Mean; SD** | **% score 9 or 10** |
|  | 1.can effectively offer PrEP care | 7.9; 2.78 | 57.4% |
|  | 2. can provide counseling to people on PrEP | 7.8;2.75 | 54.6% |
|  | 3. can effectively use the algorithms to identify people for PrEP | 7.38;3.03 | 48.5% |
|  | 4. can offer PrEP care if I have a clear protocol at hand | 8.5;2.38 | 72.1% |
|  | 5 . can collaborate effectively with colleagues in offering PrEP in my clinic | 8.5;2.5 | 74.5% |
| **Total scale** | Higher scores = more confidence about providing PrEP care- higher self-efficacy | 37.7;12.9;(1/50) |  |
| **C Professional role/compatibility** |  | **Mean; SD** | **% agree and completely agree** |
|  | 1.I see my values reflected in the implementation of PrEP | 0.70;1.05 | 59.4% |
|  | 2.Providing PrEP care will be compatible with my work in the clinic | 1.27;0.79 | 89.2% |
|  | 3. I see the values of the clinic reflected in the implementation of PrEP | 0.86;0.96 | 66.8% |
|  | 4. PrEP will be a very good fit in my clinic | 1.30;0.81 | 88.3% |
|  | 1.PrEP is easy to implement | 0.70;1.10 | 59.4% |
| **Total scale** | Higher score = more positive attitudes regarding the compatibility of PrEP with the professional role | 4.86;3.56;(-7/10) |  |
| **D Social influences** |  | **Mean; SD** | **% agree and completely agree** |
|  | 1.Many of my colleagues will approve that I offer PrEP care | 1.11;0.78 | 78.4% |
|  | 2.Many colleagues think that it will be important for me to offer PrEP care | 0.65;0.89 | 53.1% |
|  | 3.In the clinic there are many people motivated to offer PrEP care | 0.57;0.84 | 51.3% |
|  | 4.I think my colleagues would support the implementation of PrEP in the clinic | 0.97;0.75 | 75.7% |
|  | 5. If I offer PrEP care I will receive recognition from professionals who are important to me | 0.26;1.03 | 32.4% |
| Total scale | Higher scores =more positive influence of colleagues regarding PrEP | 3.56;3.14;(-4/10) |  |
| **G. Beliefs about consequences** |  | **Mean; SD** | **% agree and completely agree** |
|  | *1. Providing PrEP care is not worth it* | -1.37;0.85 | 4.4% |
|  | *2. Providing PrEP care will require more time than I have* | -0.53;1.26 | 59.4% |
|  | *3. Providing PrEP care would not be a priority to me* | -0.89;0.99 | 9% |
|  | 4. If I offer PrEP care, I would obtain a financial benefit | -0.45;1.31 | 23.4% |
| Total score | Higher scores =more negative attitudes regarding the worthiness of PrEP | -2.21;3.12;-8/7 |  |

*Note: Items in italic means negative attitudes or beliefs about PrEP*

**Table 3s. Distribution of CFIR and TDF items that did not fit in the scales- sample of HIV- HCP in Colombia.**

| items | **Survey items** | **Mean, SD** |  |
| --- | --- | --- | --- |
| Characteristics of interventions | *1. PrEP would take resources that could be better used to improve access to antiretroviral medications* | -0.25;1.14 | 26.3% |
| Concerns of PrEP in populations | *2. Lack of evidence on its effectiveness for HIV prevention* | 2.5;1.20 | *22.4%* |
| Beliefs of consequences in populations | *3. I believe PrEP would result in a reduction in condom use* | 0.77;1.15 | 72.8% |
| Beliefs of consequences in populations | *4. I believe the use of PrEP will increase stigma in populations at risk* | -0.56;0.96 | 16.3% |
| Beliefs of consequences in populations | *5. PrEP would lead to the use of medications for HIV prevention (medicalization of HIV prevention)* | 1.00;0.84 | 79.0% |
| Beliefs of consequences in populations | *6. PrEP will do more harm than good if not carefully implemented* | 0.78;1.34 | 68.2% |
| Beliefs of consequences in populations | *7. I believe PrEP would increase other sexually transmitted infections.* | 0.58;1.30 | 66.7% |
| Health systems | *8. The Colombian healthcare system is not ready to support the implementation of PrEP* | 0.56;1.10 | 59.7% |
| Professional role | *9. PrEP should not be implemented in the clinic* | -0.84;1.09 | 10.8% |
| Beliefs of consequences in populations | 10. Providing PrEP care would be the most important work I could do in the clinic | -0.11;0.98 | 24.3% |
| Beliefs of consequences in populations | 11. Providing PrEP care will be a good use of my time | 0.61;1.19 | 64.0% |

*Note: Items in italic means negative perceptions about PrEP.*
