# Supplementary material for: Applying implementation science frameworks to identify factors that influence the intention of healthcare providers to offer PrEP care and advocate for PrEP in HIV clinics in Colombia: a cross-sectional study
Source: Implement Sci Commun. 2022 Mar 16;3:31. doi: 10.1186/s43058-022-00278-2 (PMC8925047; doi:10.1186/s43058-022-00278-2)
Supplement: Supplementary file 7 — Additional file 7. CIBER analysis. [file 43058_2022_278_MOESM7_ESM.pptx]

## Slide 1
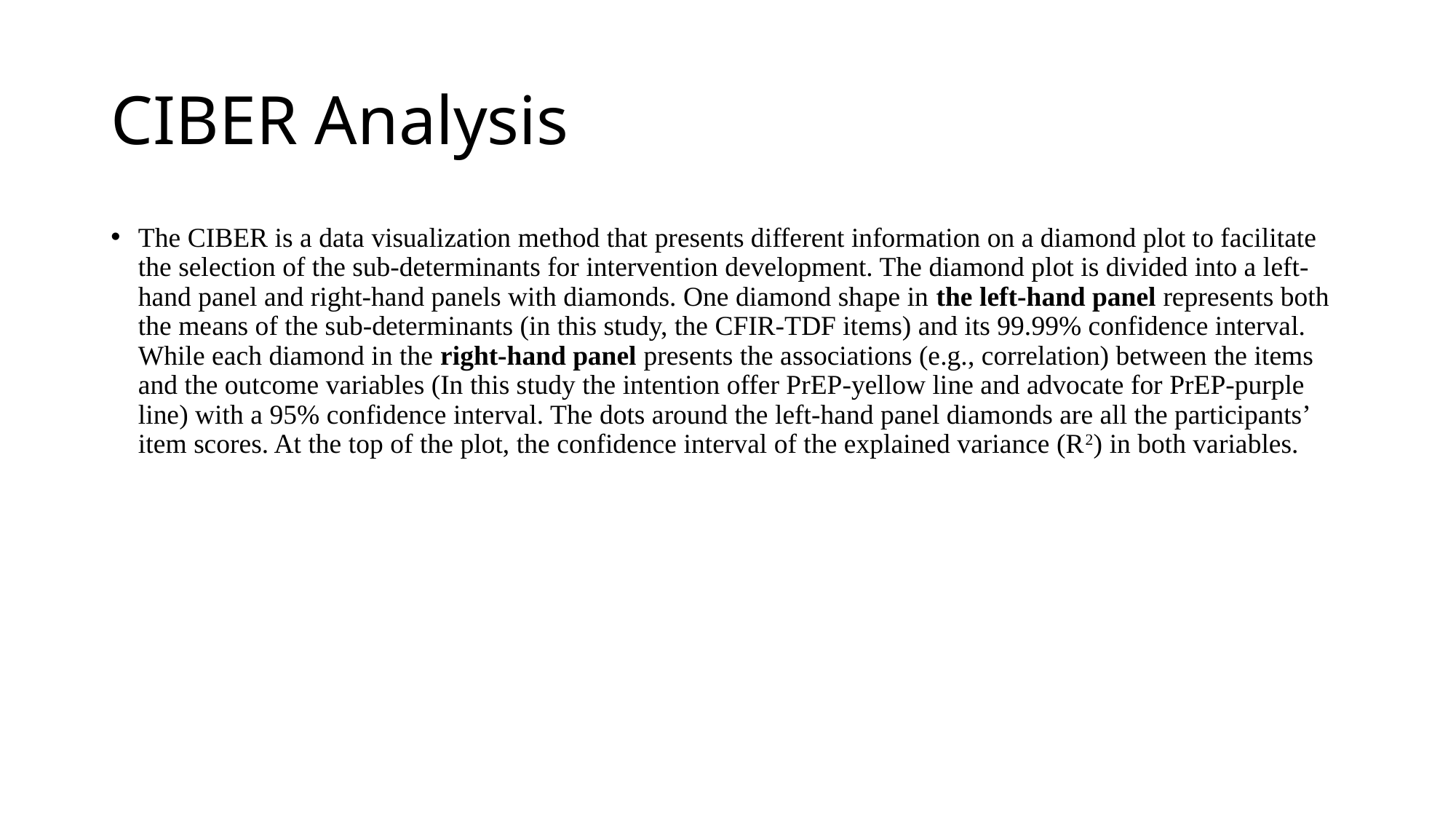

# CIBER Analysis
The CIBER is a data visualization method that presents different information on a diamond plot to facilitate the selection of the sub-determinants for intervention development. The diamond plot is divided into a left-hand panel and right-hand panels with diamonds. One diamond shape in the left-hand panel represents both the means of the sub-determinants (in this study, the CFIR-TDF items) and its 99.99% confidence interval. While each diamond in the right-hand panel presents the associations (e.g., correlation) between the items and the outcome variables (In this study the intention offer PrEP-yellow line and advocate for PrEP-purple line) with a 95% confidence interval. The dots around the left-hand panel diamonds are all the participants’ item scores. At the top of the plot, the confidence interval of the explained variance (R2) in both variables.

## Slide 2
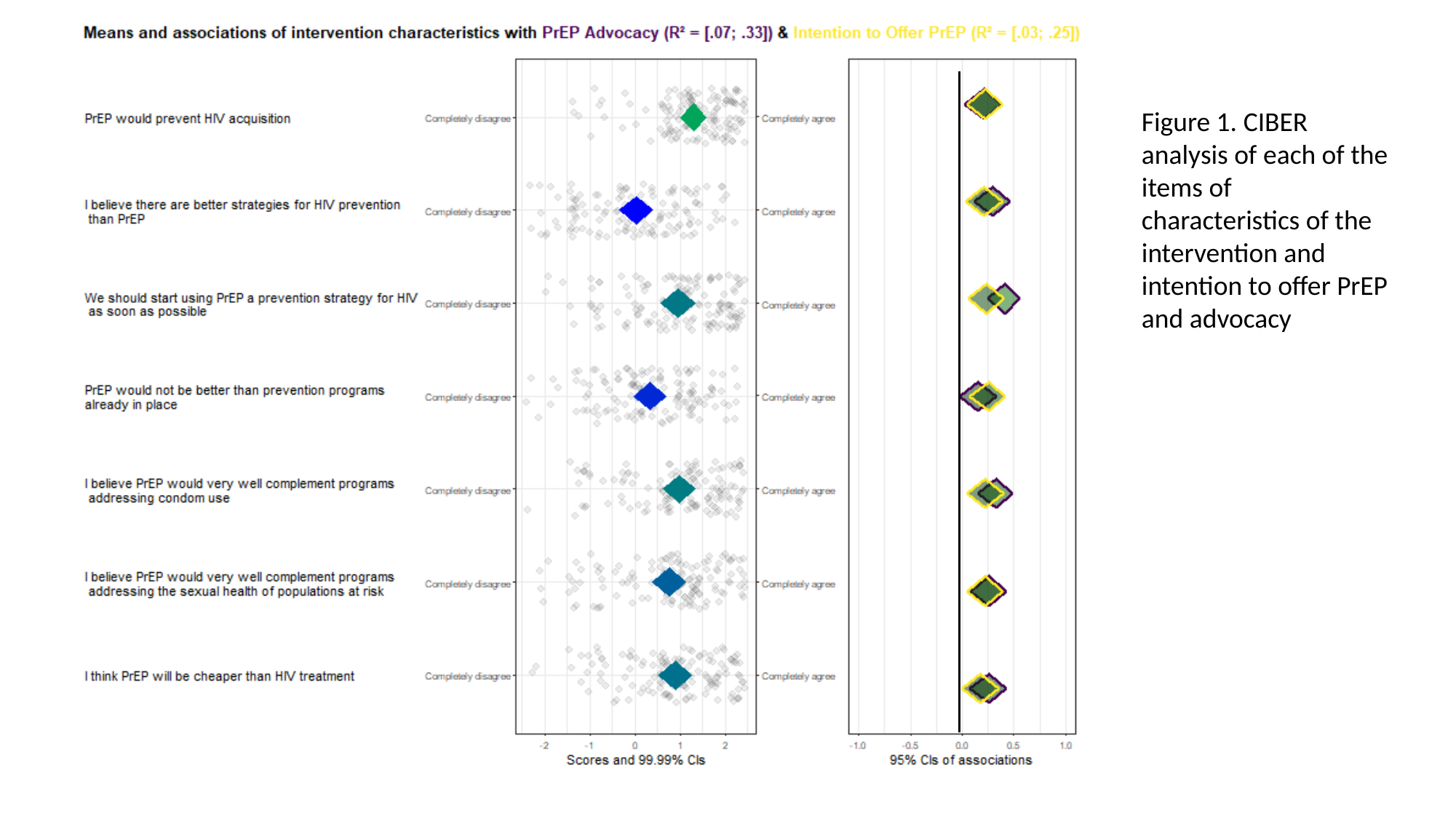

Figure 1. CIBER analysis of each of the items of characteristics of the intervention and intention to offer PrEP and advocacy

## Slide 3
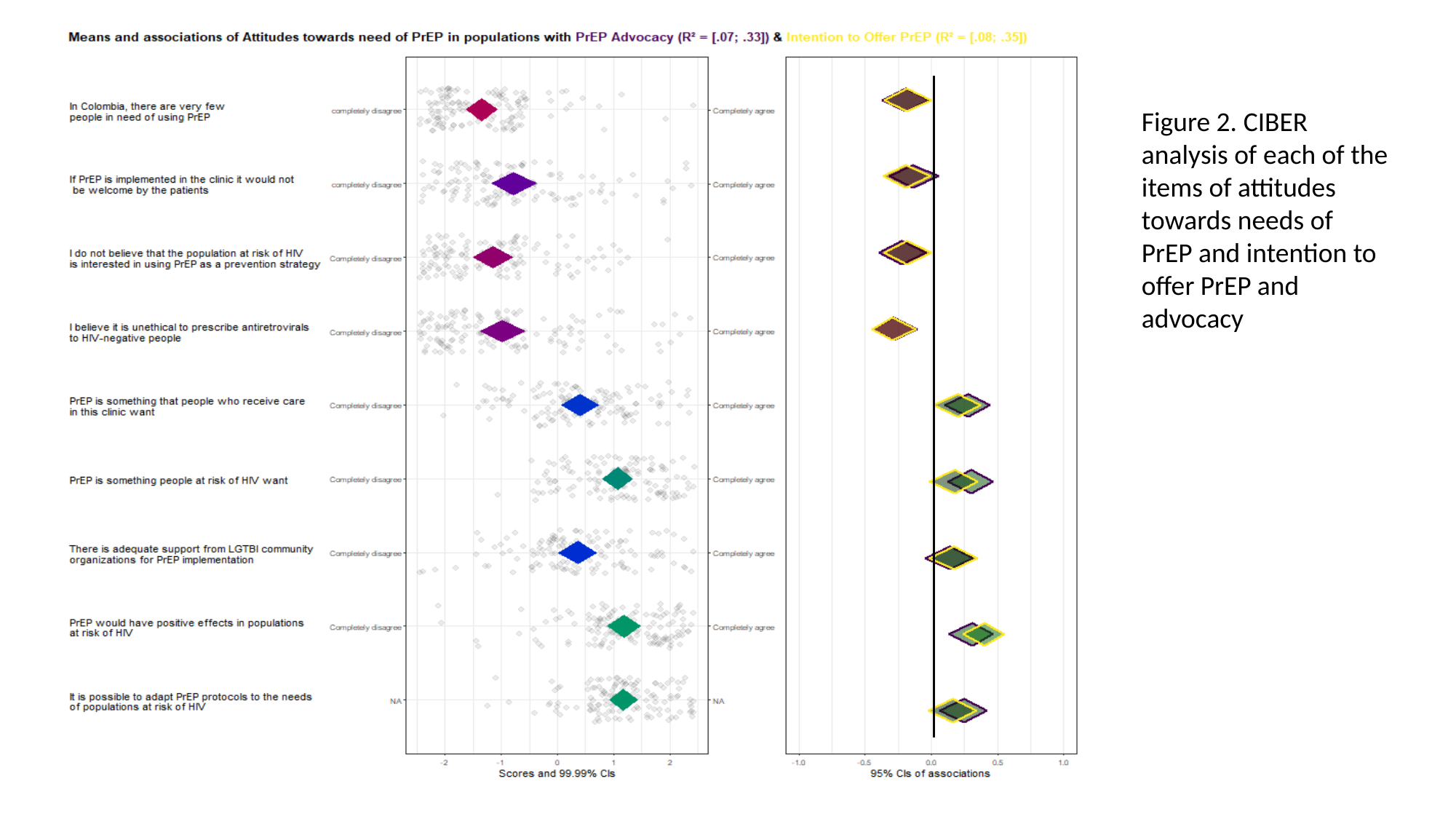

Figure 2. CIBER analysis of each of the items of attitudes towards needs of PrEP and intention to offer PrEP and advocacy

## Slide 4
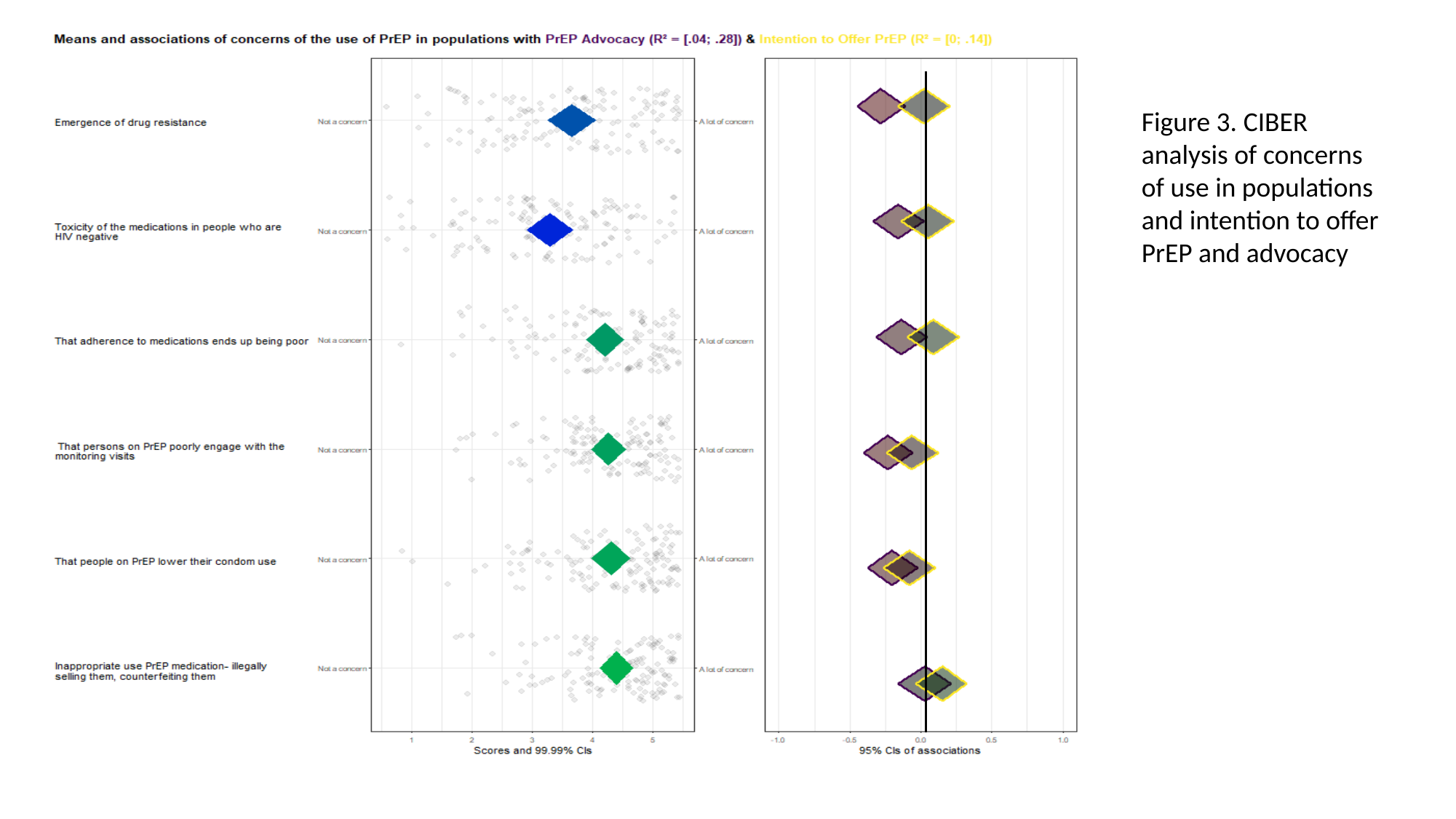

Figure 3. CIBER analysis of concerns of use in populations and intention to offer PrEP and advocacy

## Slide 5
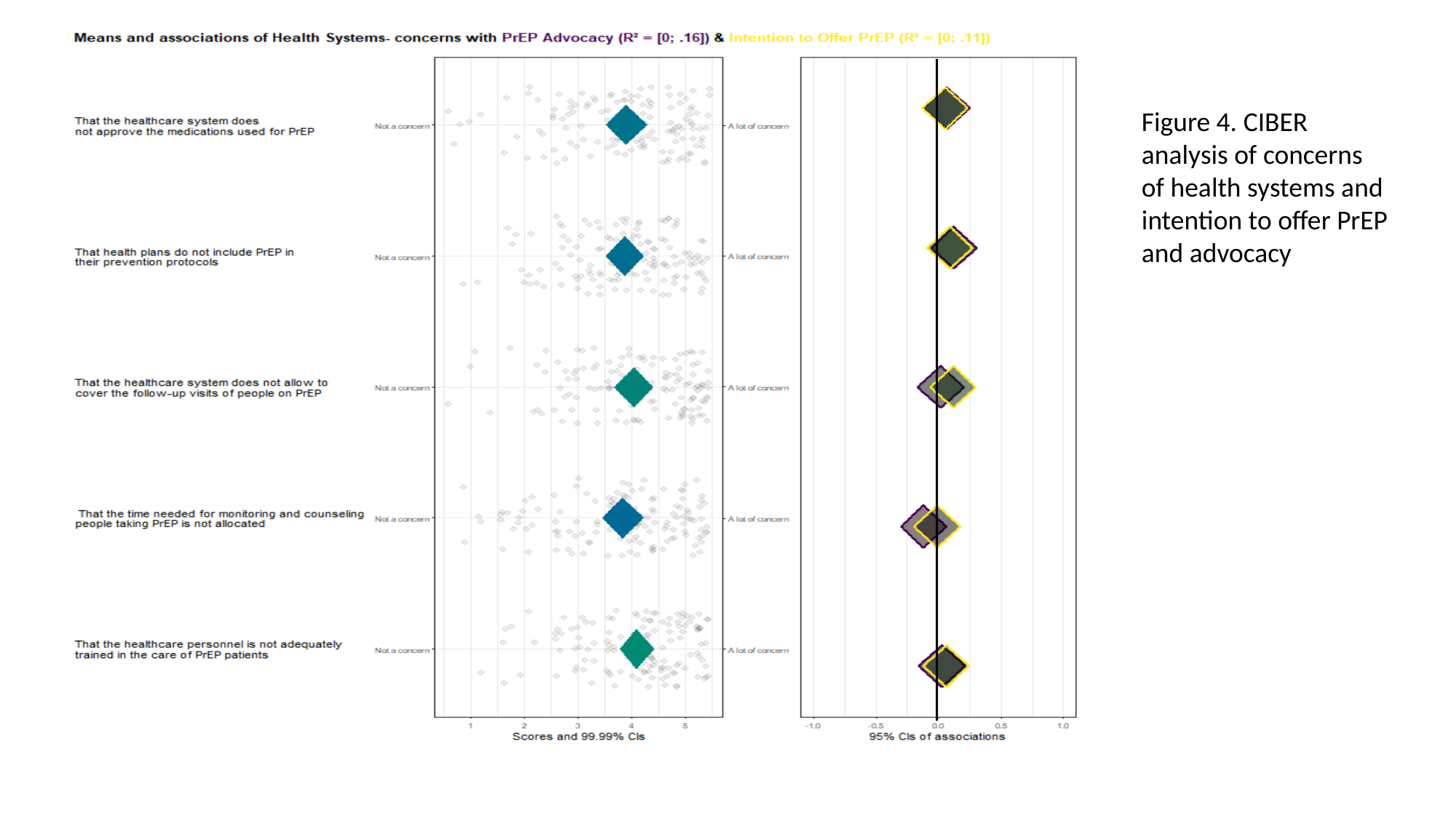

Figure 4. CIBER analysis of concerns of health systems and intention to offer PrEP and advocacy

## Slide 6
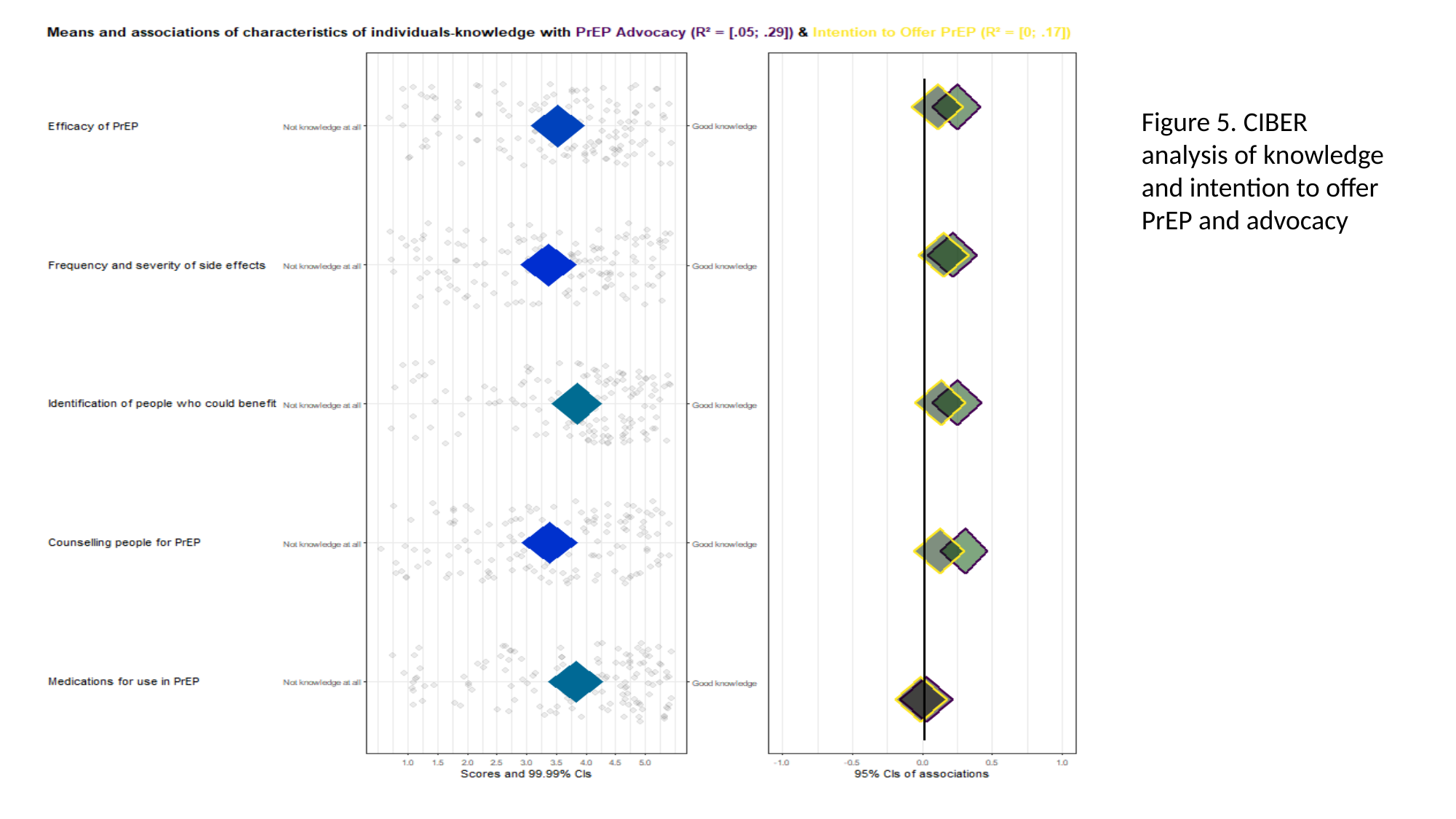

Figure 5. CIBER analysis of knowledge and intention to offer PrEP and advocacy

## Slide 7
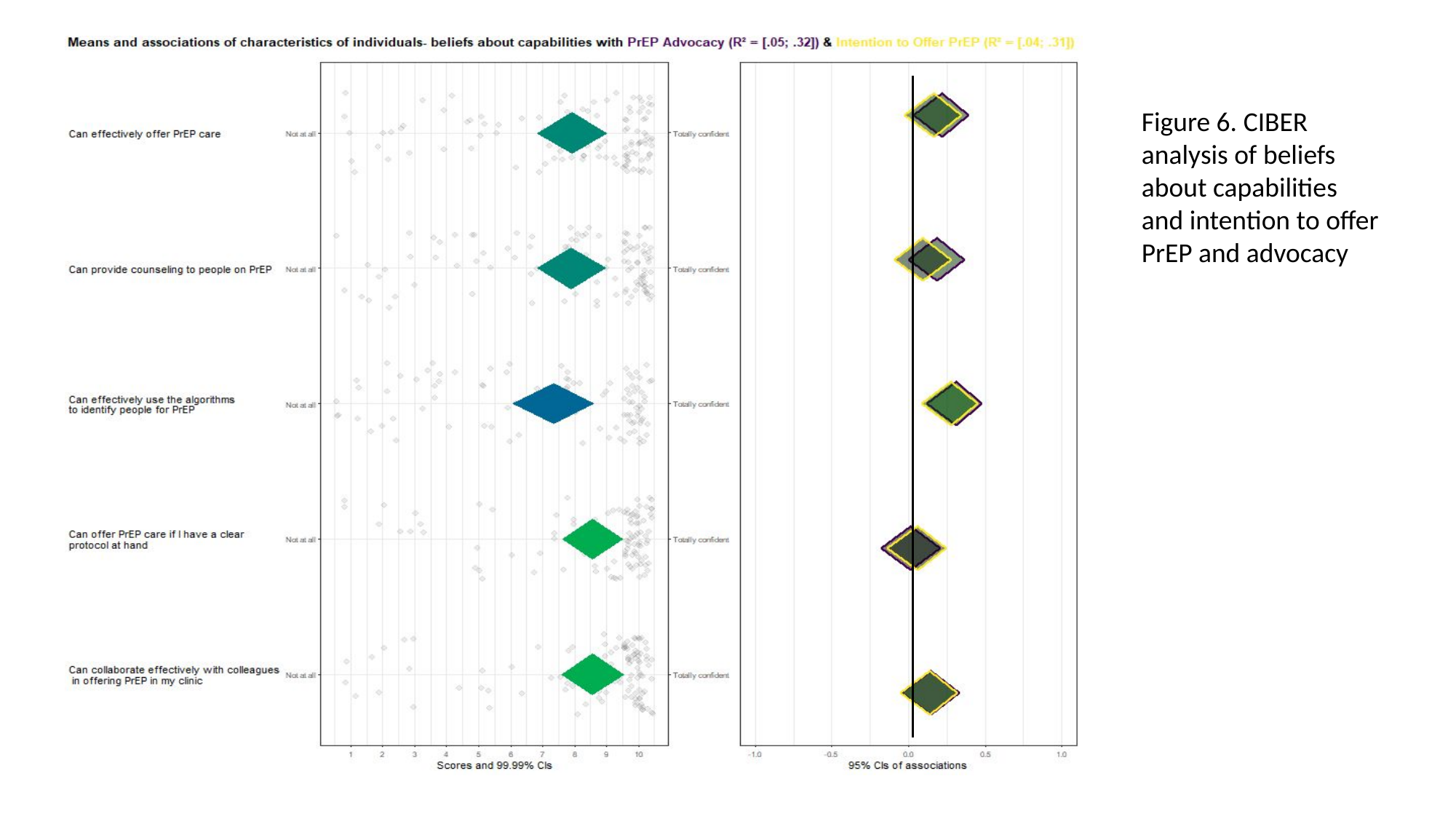

Figure 6. CIBER analysis of beliefs about capabilities and intention to offer PrEP and advocacy

## Slide 8
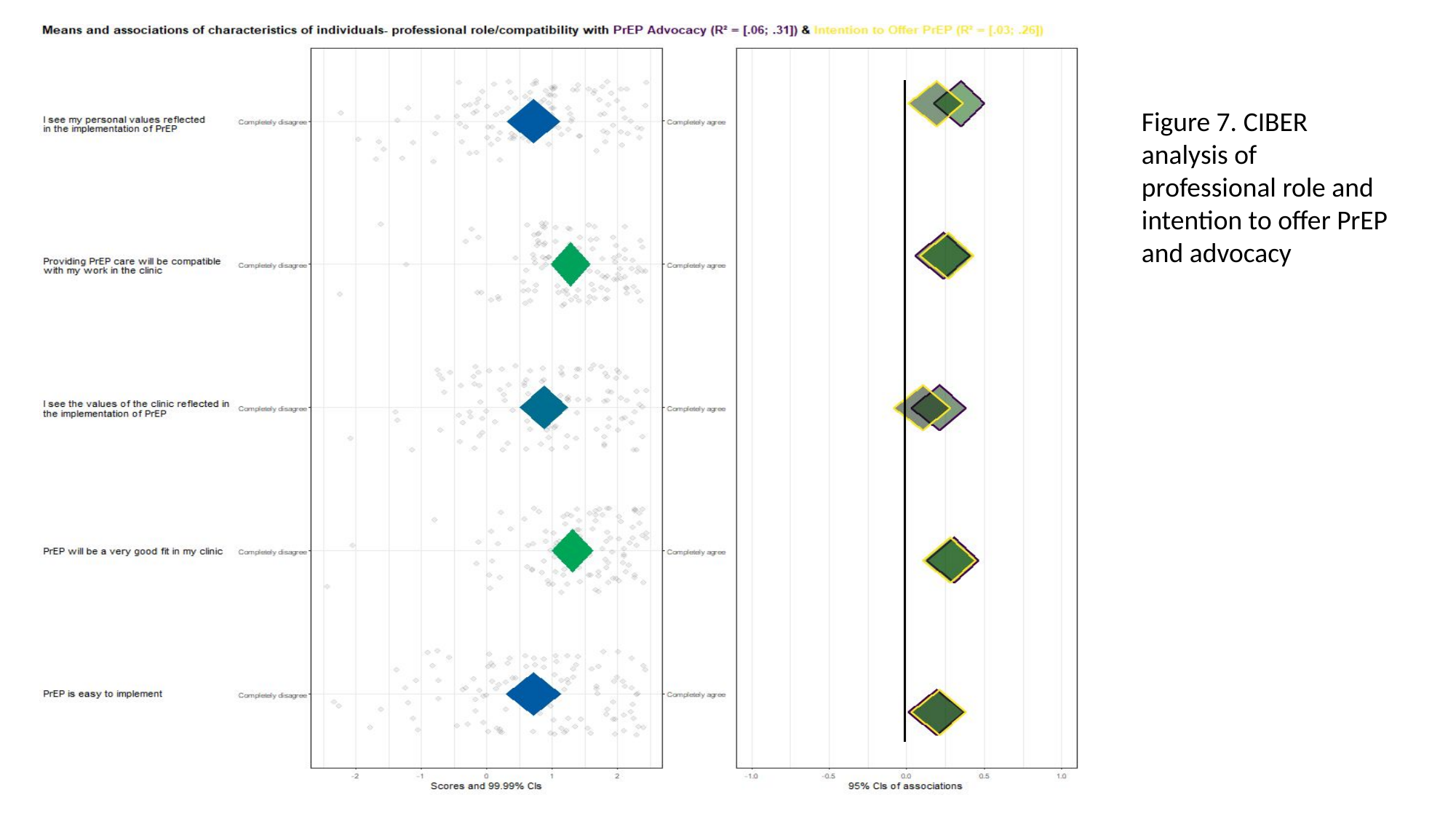

Figure 7. CIBER analysis of professional role and intention to offer PrEP and advocacy

## Slide 9
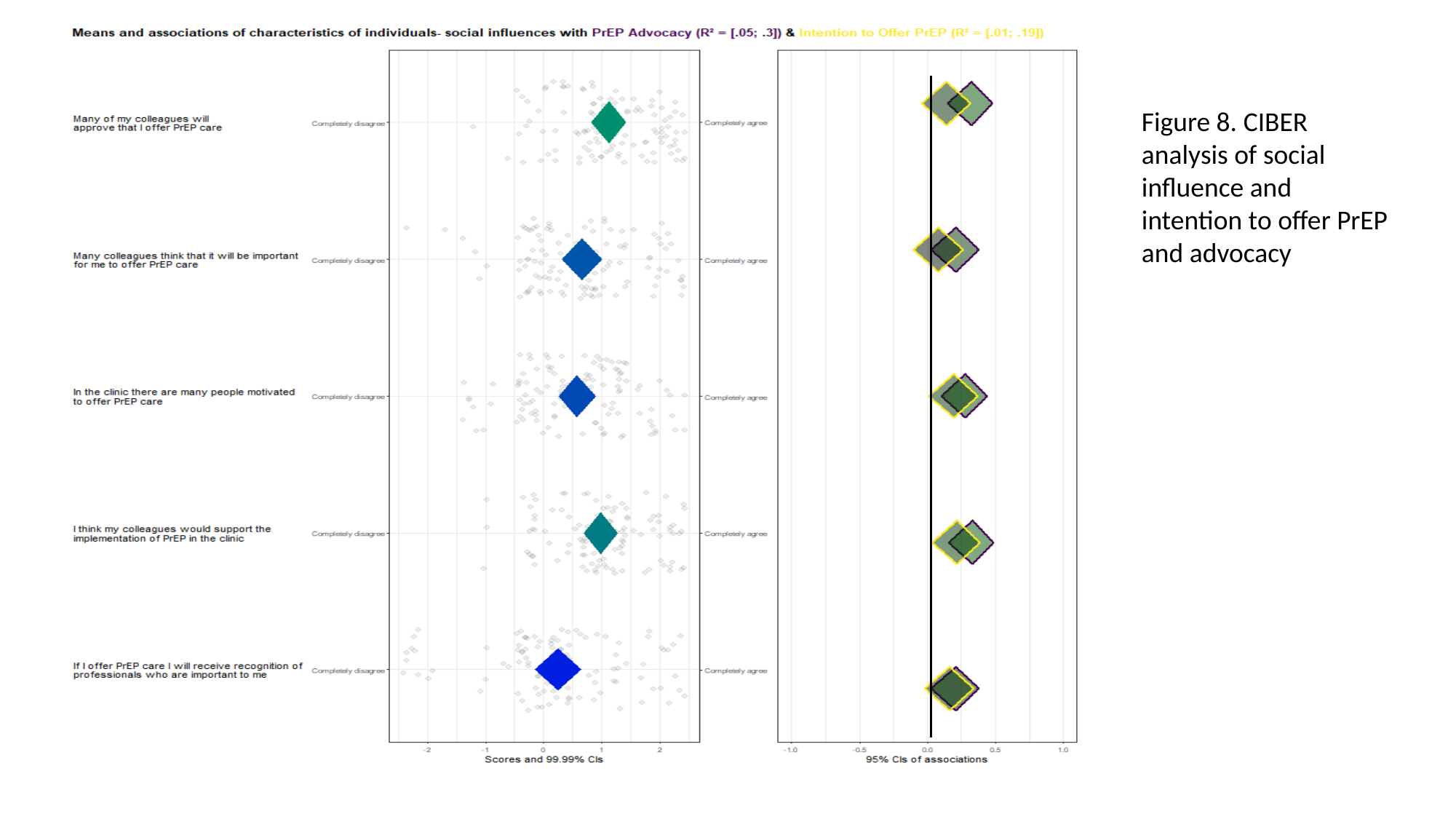

Figure 8. CIBER analysis of social influence and intention to offer PrEP and advocacy

## Slide 10
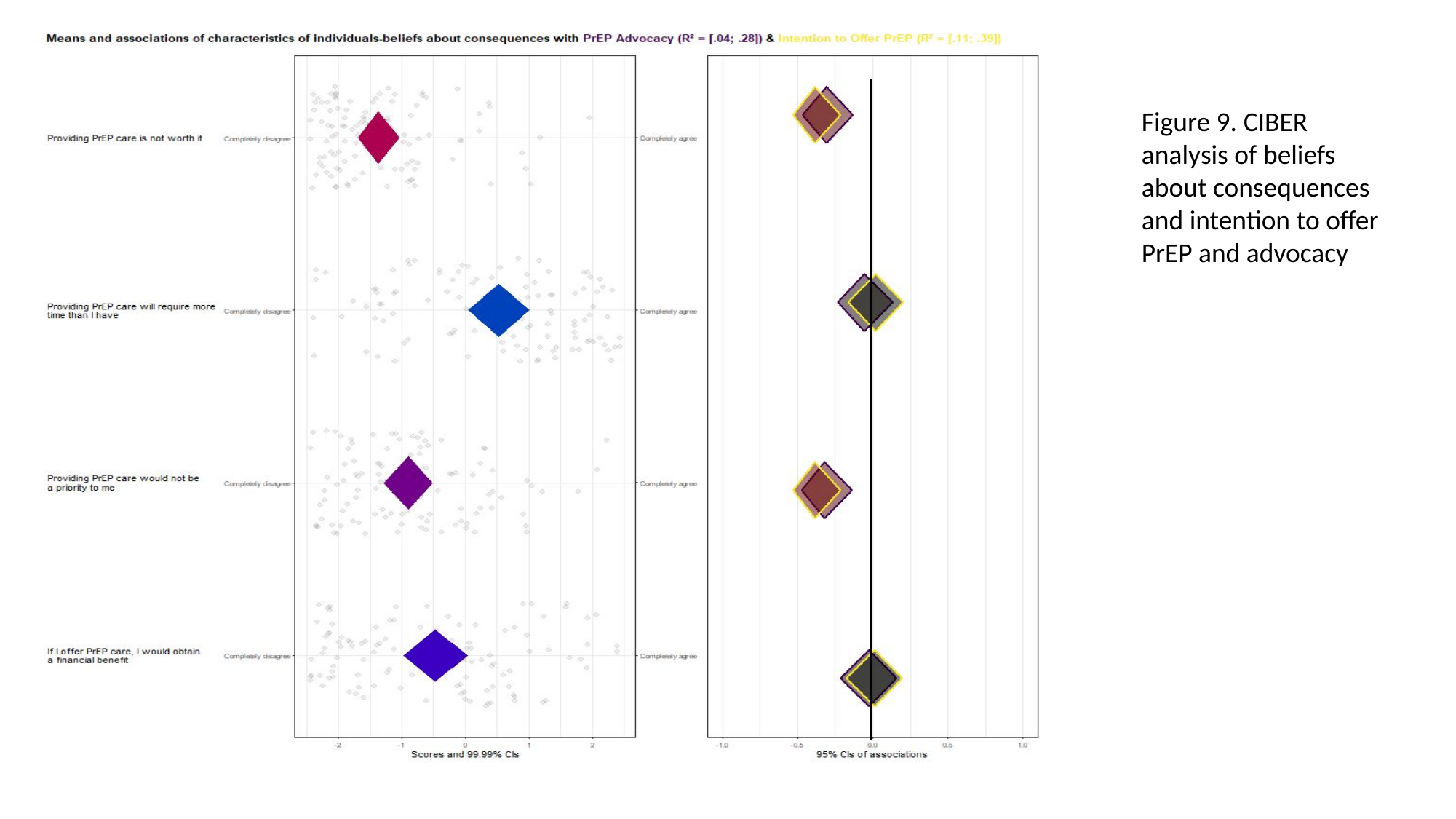

Figure 9. CIBER analysis of beliefs about consequences and intention to offer PrEP and advocacy

## Slide 11
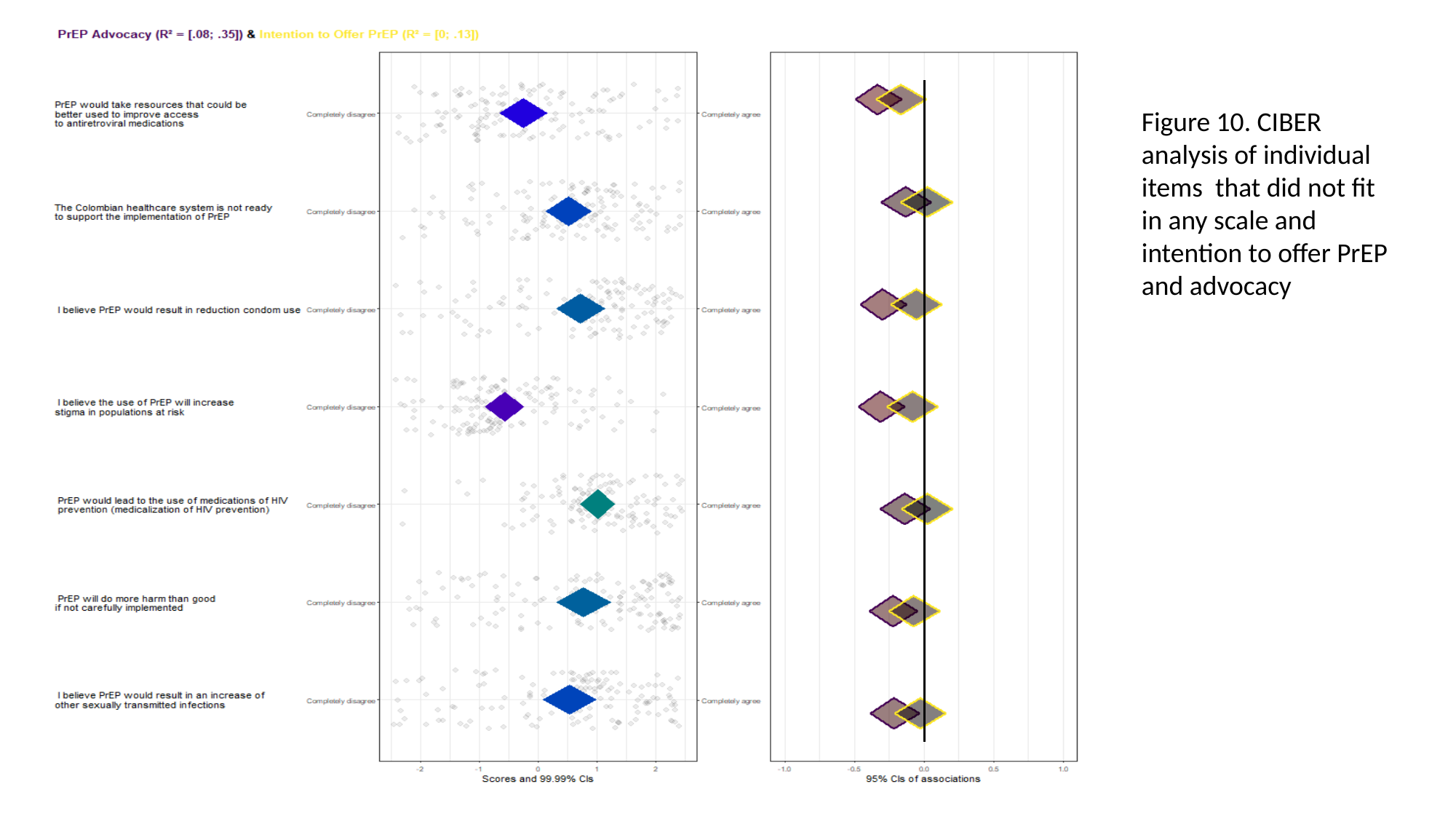

Figure 10. CIBER analysis of individual items that did not fit in any scale and intention to offer PrEP and advocacy

## Slide 12
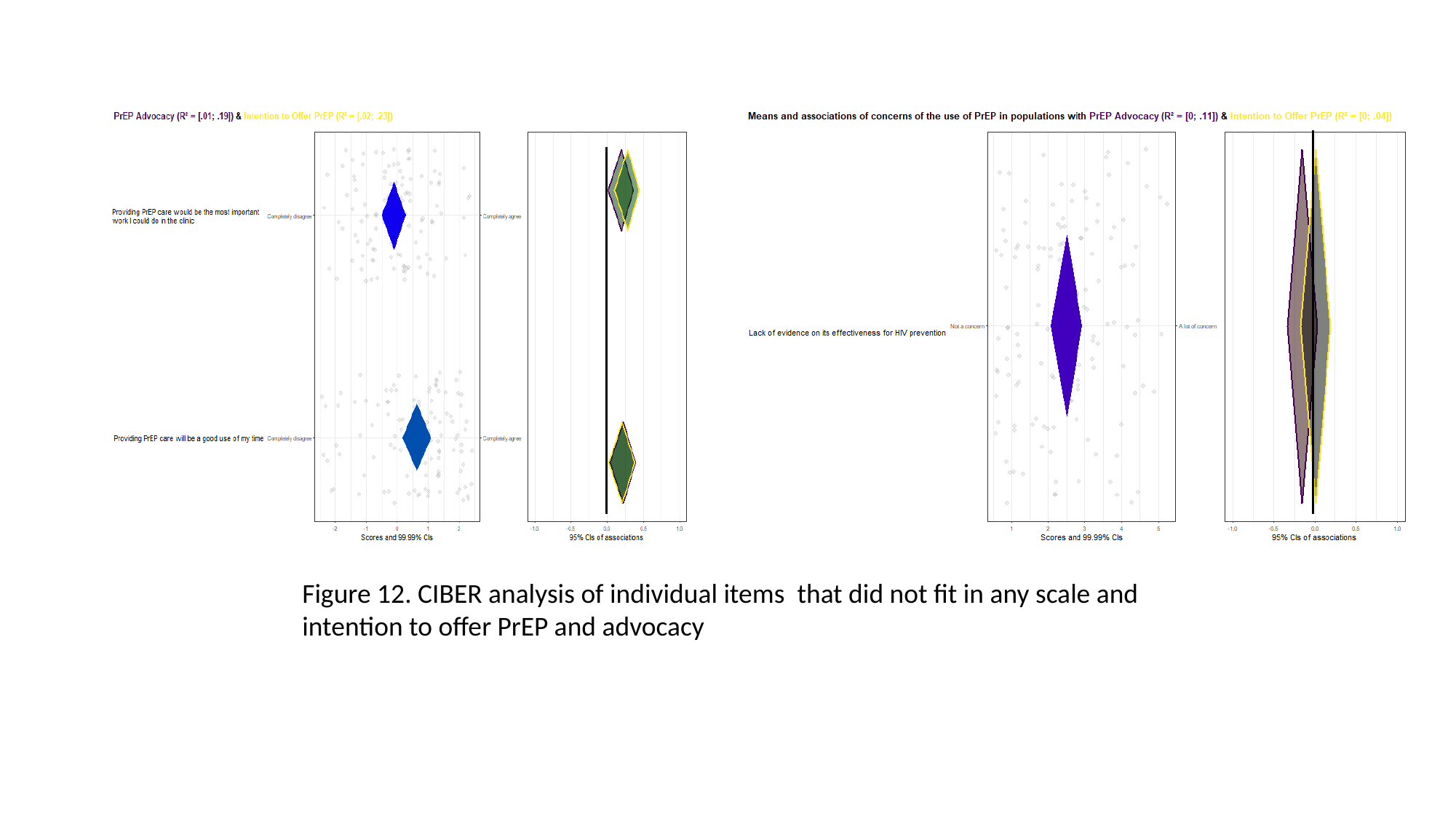

Figure 12. CIBER analysis of individual items that did not fit in any scale and intention to offer PrEP and advocacy
